# Mitochondrial transfer/transplantation: an emerging therapeutic approach for multiple diseases

**DOI:** 10.1186/s13578-022-00805-7

**Published:** 2022-05-19

**Authors:** Zonghan Liu, Yi Sun, Zhengtang Qi, Lu Cao, Shuzhe Ding

**Affiliations:** 1 School of Physical Education & Health Care, East China Normal University, Shanghai, China; 2 Key Laboratory of Adolescent Health Assessment and Exercise Intervention of Ministry of Education, East China Normal University, Shanghai, China

**Keywords:** Mitochondrial transfer, Mitochondrial transplantation, Stem cell, Energy metabolism, Ageing, Tissue injury, mtDNA mutations and deletions, Cancer therapy

## Abstract

Mitochondria play a pivotal role in energy generation and cellular physiological processes. These organelles are highly dynamic, constantly changing their morphology, cellular location, and distribution in response to cellular stress. In recent years, the phenomenon of mitochondrial transfer has attracted significant attention and interest from biologists and medical investigators. Intercellular mitochondrial transfer occurs in different ways, including tunnelling nanotubes (TNTs), extracellular vesicles (EVs), and gap junction channels (GJCs). According to research on intercellular mitochondrial transfer in physiological and pathological environments, mitochondrial transfer hold great potential for maintaining body homeostasis and regulating pathological processes. Multiple research groups have developed artificial mitochondrial transfer/transplantation (AMT/T) methods that transfer healthy mitochondria into damaged cells and recover cellular function. This paper reviews intercellular spontaneous mitochondrial transfer modes, mechanisms, and the latest methods of AMT/T. Furthermore, potential application value and mechanism of AMT/T in disease treatment are also discussed.

## Introduction

Mitochondria are essential organelles that not only serve as energy factories, but also have functions extending to cell signalling. There is much evidence that mitochondria are semiautonomous, semiself-replicating, highly dynamic organelles, endowed with their own circular, double-stranded DNA molecule that spans 16.6 kb and intramitochondrial replication and translational machinery [1]. Mitochondria continuously undergo fission, fusion, and motility, which are collectively termed mitochondrial dynamics [2]. Time-lapse videography of living cells captures spectacular intracellular mitochondrial movement, and this movement promotes mitochondrial connections to form a dynamic mitochondrial network [3]. Intracellular mitochondrial movement is of great importance for cellular functions. For example, the mitochondrial movement in neurons is finely tuned to meet the local energy requirements and calcium buffering [4]. During lymphocyte migration, mitochondria specifically redistribute to and concentrate at the uropods, facilitating lymphocyte polarization and migration [5].

In addition to intracellular mitochondrial movement, intercellular mitochondrial transfer between mammalian cells has recently been discovered. Intercellular mitochondrial transfer can be regarded as an extension of intracellular mitochondrial movement or intercellular communication, which undoubtedly increases the mtDNA content of the recipient cells and restores the respiration and survival of the recipient cells. The intercellular mitochondrial transfer phenomenon has been observed in vitro and in vivo, both physiologically and pathophysiologically, and among various cells, even cancer cells [6–8]. In the field of stem cells, cumulative evidence has shown that mitochondrial transfer contributes to stem cell-triggered repair of damaged cells [9]. More nuanced research through mitochondrial and cell staining and microscopy observations has shown that cell transfer intact mitochondrial, rather than purely mtDNA alone to help recipient cell mitochondrial function [10, 11]. Based on these findings, the researchers proposed the bold idea of directly transplanting entire mitochondria, rather than drugs or whole cells, into lesions to explore the rescue effects of exogenous mitochondria.

Collectively, although scientists are increasingly aware of mitochondria's dynamic and transferable capabilities of mitochondria, the unclear mechanism and effectiveness of mitochondrial transfer/transplantation therapy limit its widespread application in diseases. Based on the nascent literature on mitochondrial transfer, we address the modes of mitochondrial transfer, their mechanisms, and their roles in physiological and pathological conditions. In addition, we also discuss the existing methods of artificial mitochondrial transfer/transplantation (AMT/T) and their therapeutic effects on diseases to explore the potential applications of mitochondrial transfer and transplantation in the treatment of mitochondrial-related diseases, mitochondrial diseases, and cancer.

## Modes of intercellular mitochondrial transfer

Mitochondrial transfer is a novel and intriguing mechanism in intercellular communication. A majority of studies illustrate mitochondrial transfer through Tunnelling nanotubes (TNTs), Extracellular vesicles (EVs), and Gap junction channels (GJCs). There are some other modes of transferring mitochondria, such as cell fusion. Different modes of metastasis may be related to cell types and cell conditions. This section reviews the modes of mitochondria transfer discovered thus far and discusses synergies of these modes.

### Spontaneous mitochondrial transfer

TNTs are transient filamentary membranes connecting cells, consist of the cell membrane, f-actin, myosin, and tubulin. The width of TNTs is 50–1500 nm, which is sufficient for TNTs to transport proteins, RNA, or whole organelles such as mitochondria and endoplasmic reticulum to neighbouring cells or cells hundreds of microns away [12]. Rustom et al. initially observed TNTs in cultured rat pheochromocytoma PC12 cells, human embryonic kidney (HEK) cells, and normal rat kidney cells [13], and later, Koyanagi et al. observed that TNTs can transfer mitochondria between neonatal rat cardiomyocytes and endothelial progenitor cells [14]. These structures has been observed in immune cell tumour cells, nerve cells, and muscle cells [15], suggesting that TNTs may be a common method of communication between mammalian cells. The crucial structure in TNTs is F-actin. On the one hand, F-actin crosslinking ensures the rigidity of TNTs, conferring stability against buckling for TNT outward growth and proper protrusion length [16, 17]; on the other hand, F-actin crosslinking enables mitochondria to be transported along cytoskeleton structure of TNTs [18]. Interestingly, TNT-mediates can be unidirectional or bidirectional. Unidirectional transfer prevailingly from healthy to damaged cells [19, 20], Bidirectional mitochondrial transfer has also been reported, but the amount of mitochondria transferred is different between the two types of cells [21] and the benefits that recipient cells receive is different [22]. This suggests that TNT-mediated mitochondrial transfer is active and regulated. This regulatory mechanism requires further investigation.

EVs, including exosomes, microvesicles, and apoptotic bodies, are cell-secreted nanoscale, bilayered structure vesicles that can carry various lipids, proteins, RNA, miRNAs, and mitochondria [23, 24]. Moreover, EVs can exist in the extracellular fluid stably and participate in cell communication, cell migration, angiogenesis, and tumour cell growth as important messengers. EV-mediated mitochondrial transfer was found in multiple tissues as part of essential cellular bioprocesses. Previous studies suggested that astrocytes secrete EVs to regulate neuronal function, synaptic formation, and maintenance [25]. Hayakawa et al. found that astrocytes released mitochondrial particles and then partially absorbed them by damaged neurons to promote neuronal survival and dendrite regrowth [26]. Cardiac-resident macrophages can capture and eliminate the defective mitochondrial particles released by cardiomyocytes to support heart function and metabolism [27]. Alternatively, EV-mediated mitochondrial transfer is implicated in immune regulation. For example, airway myeloid-derived regulatory cells transfer exosomal mitochondria to the T cells, and then exosomal mitochondrial integrate with the T cells mitochondrial network and generate reactive oxygen species (ROS) [28].

GJCs are the most direct material exchange channels between two adjacent cells formed by docking their respective hemichannels (HCs). HCs are hollow tubular structure formed on the cell membrane by the oligomerization of six connexin (Cx) subunits. Notably, HCs may consist of the same Cx isotype (homomeric) or different Cxs isotypes (heteromeric). Two identical HCs docking form homotypic GJCs, and two different HCs form heterotypic GJCs [29]. This characteristic indicates that different tissues or cells can transport material by diverse GJCs or HCs. Cells seem to form homotypic GJCs preferentially. Islam et al. instilled bone marrow mesenchymal stem cells (BMSCs) expressing only CX43 into alveolar cells expressing multiple CX subunits and found that BMSCs attached only to the high expression sites of CX43 in alveolar cells [30]. However, in some cases, the two kinds of cells do not express the same CXs, but can also form heterologous GJCs to mediate mitochondrial transfer. Li et al. found that BMSCs could transfer mitochondria to oxygen–glucose deprivation (OGD)-injured motor neurons through GJCs in a transwell coculture system to promote motor neuron survival, while CX43 was expressed only in MSC cells and CX32 was expressed only in motor neurons, indicating that the two types of cells formed heterotypic GJCs composed of CX43 and CX32 [31]. When GJC inhibitors were added, mitochondrial transfer was severely impaired, suggesting that heterologous GJCs also mediated mitochondrial transfer.

Mitochondria can transfer from cell to cell due to their excellent deformability. Mitochondria vary from 0.5 to 1 µm in diameter and from 0.5 to 10 µm in length and show remarkable variation in different tissues [32]. In general, mitochondria are rod-like or spherical, but the morphology of mitochondria is regulated by continuous fusion and fission events, and mitochondria can fuse or connect to form a mitochondrial network [33]. Mitochondria exhibit significant morphological changes that allow them to pass through TNT structures and be enveloped in EVs. However, the pore size of the gap junction is only 1.5–2 nm, and only substances with a molecular weight of less than 1.5 kDa are allowed to pass through [29]. Therefore, mitochondria obviously cannot be directly trafficked intercellular through GJCs. A recent study by Alarcon-Martinez revealed that a structure composed of TNTs and one distal-side CX43 based-GJC connects two pericytes on separate capillary systems, which they named interpericyte tunnelling nanotubes (IP-TNTs), and these IP-TNTs can regulate neurovascular coupling in the living retina. They also confirmed that mitochondria were present in and travelling within IP-TNTs using time-lapse imaging. However, mitochondrial transfer between pericytes did not occur due to the restrictions of the distal side GJCs of IP-TNTs [34]. Similarly, Pinto et al. found that GBM stem-like cells (GSLCs) communicate through tumour microtubes (TMs) and TNTs structures in the culture of GSLCs tumour organoids; however, the intercellular mitochondrial transfer can only be observed in TNTs, rather than in TMs [35]. TMs are organelle-rich and thick (> 1 µm) protrusions that contain GJCs along their length. Time-lapse imaging showed that mitochondria travelled rapidly in TMs, whereas no transmembrane transfer was observed. This travel seems to only provide energy for GSLCs molecular exchange via GJCs in TMs [36].

Nevertheless, the GJC potentiator retinoid acid or inhibitor 18β-GA can significantly affect intercellular mitochondrial transfer [31]. One possibility is that GJCs mediate mitochondrial transfer through their nonclassic form. GJCs form annular gap junctions (AGJs) and are internalized in one of two adjacent cells. This internalization engulfs a small fraction of a connected cell and has been observed and well described by electron microscopy and live-cell imaging [37]. In the past, GJC internalization was thought to regulate the number of GJCs available for communication, and now evidence points to additional intercellular communication. By using 3D electron microscopy and immunogold labelling of CX43, Norris et al. found that receptor cells mitochondria into double-membrane-vesicle AGJs through CX43-based GJC internalization [38]. Of course, more follow-up research is needed. For example, whether the mitochondria contained in the AGJs in the receptor cells will degrade, escape from the AGJs, or stably exist in the AGJs and secrete their products, such as ATP, through GJCs is unknown. An exciting study demonstrated that the frequency of interaction between mitochondria and AGJs was greater than that between lysosomes and annular gap junctions [39]. This may indicate that mitochondria are packaged by AGJs and sent to recipient cells with a high probability and certain biological basis; moreover, the active interaction may indicate that internalized AGJs are not destined for degradation and are more likely to exchange material with endogenous mitochondria [31].

Another possibility is that GJCs assist in TNT-mediated mitochondrial transfer. Immunofluorescence staining showed that the structure F-actin and microtubules of TNTs overlapped with CX43 at the astrocyte-neuron contact sites [40]. Data from Drs. Osswald and Eugenin showed that CX43 is present in TNT-like structures, and the inhibition of GJCs does not prevent TNT formation but does interfere with regular communication between TNT connective cells [41]. Intriguingly, Yao et al. also observed CX43 in TNTs between human induced pluripotent stem cell-derived MSCs (iPSC-MSCs) and BEAS2B cells, and knocking down the expression of CX43 significantly affected the formation of TNTs and decreased mitochondrial transfer between the two cells [42]. Conversely, enhanced Cx43 expression promotes mitochondrial transfer from astrocytes to neurons via TNT-like structures [43]. In GSLCs, the cell subpopulations having more TMs have a higher efficiency of TNT-mediated mitochondrial transfer, even after irradiation [35]. Considering that TMs cannot transfer mitochondria, TMs may contact cells and conduct more signal exchanges through GJCs, thereby accelerating the formation of TNTs and the occurrence of mitochondrial transfer. A similar study showed that CX43-based GJCs mediate mouse BMSC (mBMSC) attachment to alveolar epithelial cells, and this attachment facilitates the formation of TNTs between the two types of cells and the vesiculation of mBMSCs [30]. These findings collectively illustrate that CX43-based GJCs are involved in TNT-mediated mitochondrial transfer.

At present, the model/structure of the combination of TNTs and CX43-based GJCs in intercellular communication is still ambiguous and not uniformly described in different studies. For example, some TNTs are open-ended, some are closed, and some are closed at one end. In general, the closed-ended TNTs facilitate small-molecule exchange between cells through CX43-based GJCs [44, 45]. Whereas immunostaining results show that CX43 can be located in the middle of TNTs and also on the mitochondrial membrane [42, 46], CX43 may not function as the composition of GJCs but as independent functional proteins. Basheer et al. found that CX43 could interact with actin and tubulin, and the exogenous expression isoform of the C-terminal tail of CX43 stabilized F-actin without affecting the expression of actin [47]. Therefore, the role of CX43/GJCs on TNTs and the way it helps mitochondrial transfer is incompletely understood. In future studies, in addition to the observation of CX43 and TNTs by accurate fluorescence and superresolution microscopy, the membrane structure of the contact site between TNTs and cells as well as the localization and mode of action of CX43 expressed on TNTs deserve more attention.

### Nontraditional mitochondrial transfer

**Fig. 1:**
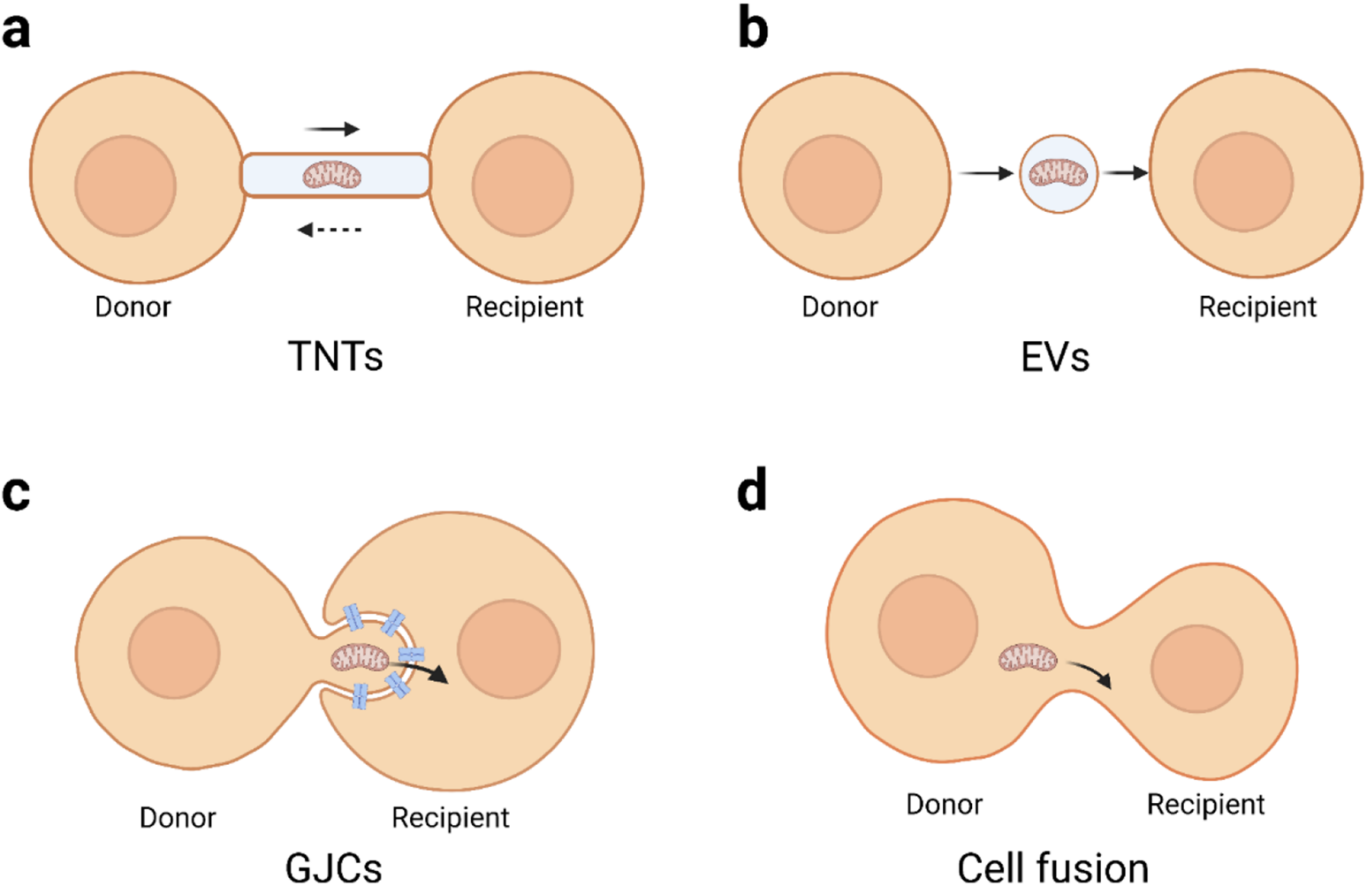
A simplified visualization of intercellular mitochondrial transfer modes. **a** Mitochondria are transported between cells via the TNT structure, and this transport is bidirectional. **b** The mitochondria-containing EVs bud off/are secreted from the donor cells and then uptake by recipient cells. **c** The mode of GJC-mediated mitochondrial transfer is ambiguous. Nonclassic form AGJs are plausible: mitochondria exist in the protrusions of donor cells, and the recipient cells connect to the donor cells through invaginating GJCs. Then the recipient cell internalizes GJCs and absorbs mitochondria. **d** Cell fusion can be spontaneous or artificial

Mitochondria can also be transferred in nonclassic ways, such as through cell fusion [48], synaptosomes [49], and dendritic networks [50]. Cell fusion can occur spontaneously in the body or be induced artificially. Spontaneous cell fusion, mainly stem cells with somatic cells, has been observed in various tissues and organs, such as skeletal muscle and the liver, lungs, heart, intestines, skin, brain, and retina, and is considered to contribute to the reprogramming of somatic cells and the regeneration of tissues [51]. Wada et al. described a Sendai virus envelope-based method that can allow two separate cells to fuse via a narrow cytoplasmic connection. Then, they could control the length of the cytoplasmic connection to achieve quantitative control of mitochondrial transfer rates [52]. Acquistapace et al. found that cell fusion may be partial rather than permanent through the coculture of cardiomyocytes and human pluripotent adipocyte stem cells. This partial cell fusion can allow the exchange of substances and mitochondria and promote cardiomyocyte reprogramming [48]. Furthermore, the result of Acquistapace et al. also showed that, permanent cell fusion and synkaryons are rarely formed under coculture conditions [48]. Additionally, Gao et al. demonstrated that mitochondria can transfer between bone cells through dendrite networks [50]; while, Picone et al. demonstrated that mitochondria can be delivered via synaptosomes. Figure 1 summarizes the different modes of mitochondrial transfer.

### Synergistic modes of mitochondrial transfer

The modes of mitochondrial transfer have highly selective and change with cell type and culture condition [53]. Inhibition of TNTs significantly reduced mitochondrial transfer from human multipotent adipose-derived stem cells to cardiomyocytes, while inhibition of GJCs had no effect on mitochondrial transfer [54]. Data from Yan et al. shows that MSCs transfer mitochondrial to ischaemia–reperfusion (IR)-injured HUVECs through TNTs rather than cell fusion and the paracrine function, and MSCs failed to rescue the injured HUVECs when the TNTs were blocked [20]. In vitro studies showed that MSCs transfer mitochondria directly to post-OGD motor neurons through TNTs or direct contact indirect coculture conditions, while transferring mitochondria via secrete particles to VSC4.1 motor neurons in indirect coculture condition [31]. Tumor cells preferentially use TNT to mediate mitochondrial transfer [55]. Marlein et al. cocultured Multiple myeloma cell lines with BMSCs, and then treated cells with TNT and endocytosis inhibitors; TNT inhibitors reduced mitochondrial transfer to multiple myeloma cell lines by up to 46.19%, whereas there was no significant reduction observed with endocytosis inhibitors [53]. Moreover, there was no mitochondrial transfer from BMSC to malignant plasma cells when multiple myelomas were cultured in indirect coculture conditions [53].

There are also cases where modes of mitochondrial transfer are synergetic. As mentioned above, CX43-based GJCs may contribute to the formation of TNTs. Live optical studies revealed that mitochondrial transfer from mBMSCs to the alveolar epithelium occurred through a complex process. mBMSCs first attach alveolar epithelium by forming CX43-based GJCs and then released mitochondria-containing microvesicles, finally mitochondria-containing microvesicles were engulfed by the epithelium [30]. After cocultivating various tissue-derived MSCs with lung epithelial cells and using different molecular inhibitors to inhibit endocytosis in epithelial cells and inhibit the formation of microtubules and gap junctions between cocultured cells, the efficiency of mitochondrial transfer significantly decreased to varying degrees. Moreover, when all inhibitors were added, the mitochondrial transfer efficiency was suppressed to a greater extent, indicating that these approaches were synergistic [56].

## AMT/T

According to the endosymbiosis hypothesis, mitochondria are derived from the ancestor of α-proteobacteria. This endosymbiont steadily lost its autonomy as it became integrated into the host and then evolved into organelles [57, 58]. As the phenomenon of intercellular mitochondrial transfer is revealed, researchers have assumed that mitochondria may still retain the ability to invade cells. They have explored various methods to transfer mitochondria to the recipient cell artificially.

### AMT/T in vitro

The earliest AMT/T can be traced back to 1982. Clark and Shay extracted purified mitochondria, including antibiotic-resistant genes, and then mixed the purified mitochondria with antibiotic-sensitive cells. Subsequently, mitochondria were internalized by cells, thereby increasing the cell antibiotic-resistant ability [59]. This coincubation transfer method continues to the present. However, the coincubation method is limited by the endocytosis effect of recipient cells. Currently, various methods have been used to improve the efficiency of mitochondrial transfer to cells, for example, using synthetic liposomes [60] or the cell-penetrating peptide Pep-1 [61], adding additional centrifugation and a thermic shock step during coincubation (MitoCeption) [62], and exploiting anti-TOM22 magnetic beads and magnetic plate to form magnetomitotransfer [63].

In addition, Kim et al. transfer mitochondria into the cells by simply centrifuging mitochondria and cell suspension at 1500×*g* for 5 min at 4 °C [64]. This method duplicated the key steps of MitoCeption, namely centrifugation and thermic shock. However, in this method, the cells were rinsed with PBS after centrifugation, which means the excess mitochondria were removed after centrifugation; the cells do not have to undergo additional mitochondria coincubation [64]. Wada et al. used a microfluidic device combined with the Sendai virus to fuse two single living cells through a microtunnel and achieved quantitative control of mitochondrial transfer, including single mitochondrial transfer [52].

However, these studies rarely focused on how long the transferred exogenous mitochondria can be preserved. Recent studies by Pour et al. and Teitell’s group have shown that the coincubation of exogenous mitochondria can significantly improve recipient cell mitochondrial respiration and cellular bioenergy in the short term. However, this effect and exogenous mtDNA rapidly disappeared with time and cell passage [65, 66]. Teitell’s group developed a photothermal nanoblade to stably deliver isolated mitochondria into ρ0 cells (a type of mtDNA-deficient cell line) to resolve the exogenous mitochondrial stable retention issue. However, the nanoblade was low throughput and had equivocal effects (one of the three clones did not recover its metabolic activity) [67]. They subsequently invented Mitopunch, a versatile mitochondrial transfer technology that can permanently transfer mitochondria from various sources to multiple types of receptor cells and form stable isolated mitochondrial recipient (SIMR) cells with unique mtDNA-nDNA combinations [68]. Mitopunch was also high throughput and rapid (generating a large population of stable mtDNA-nDNA combination clones within two weeks) [68]. They recently compared Mitopunch, Mitoception, and coincubation and found that Mitoception was greater than coincubation in comparing mitochondrial transfer efficiency after two-hour delivery, while coincubation was greater than Mitopunch [69]. However, coincubation produced almost no SIMR; Mitopunch or Mitoception could lead to dozens of immortalized SIMR clones, but only Mitopunch could produce replication-limited nonimmortality human SIMR clones, and Mitopunch had a better saving effect on mitochondrial respiration of ρ0 cells [69].

Mitochondrial retention may be related to the interplay between mitochondrial and nuclear genomes. Dawson et al. transferred chloramphenicol-resistant mitochondria to chloramphenicol-sensitive, metabolically impaired ρ + mouse cybrid cells by Mitopunch, following by uridine deficient and CAP-supplemented media selection, found that the chloramphenicol-resistant mtDNA was stably retained in recipient cells with mismatched nDNA and mtDNA genomes and replaced the endogenous mtDNA. In comparison, recipient cells with matched nDNA and mtDNA genomes did not enable the retention of the transferred mtDNA [66]. The results indicate that nDNA genomes play a crucial role in mitochondrial preservation.

**Fig. 2:**
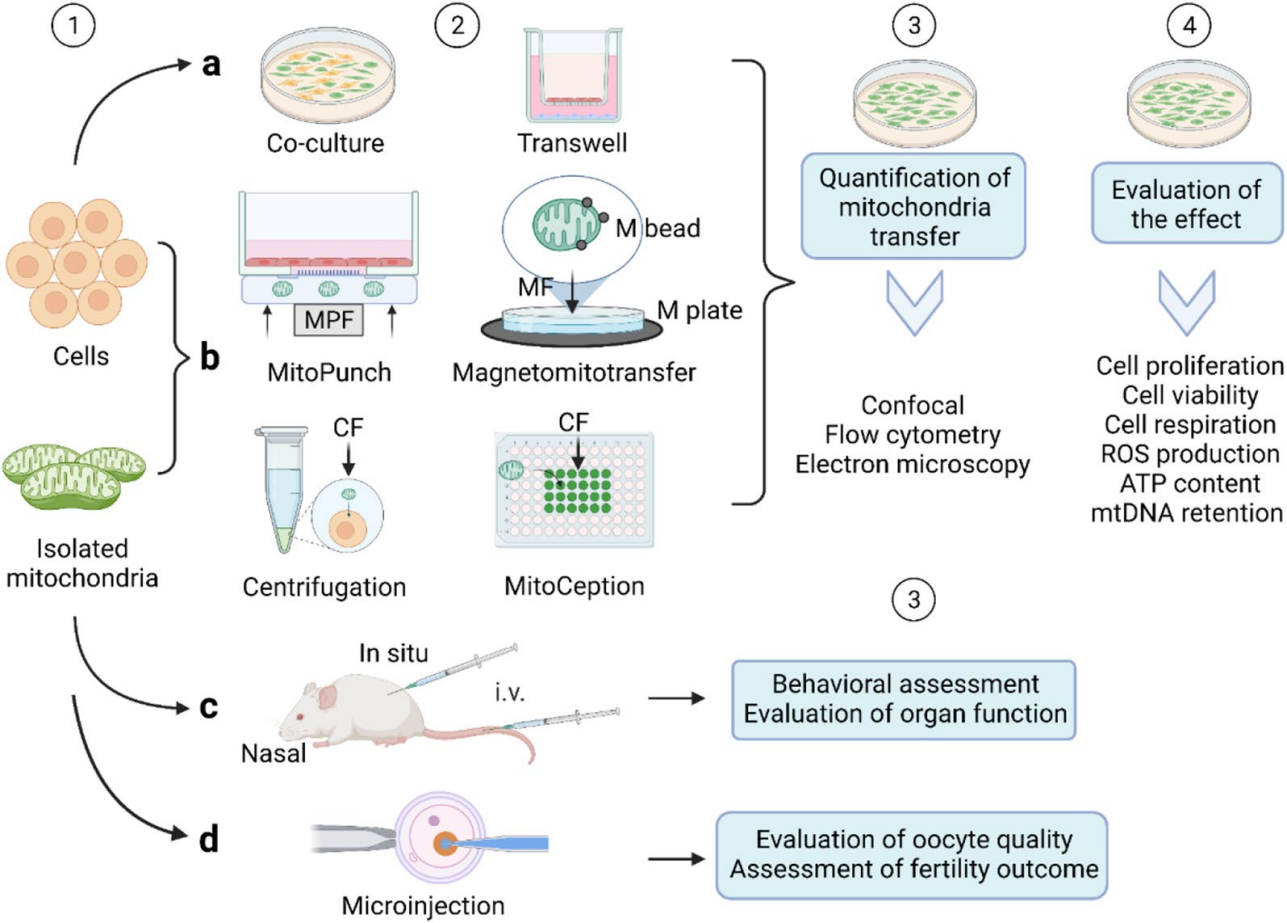
A flow diagram of AMT//T. Step 1, the mitochondria are isolated and the cells are prepared. Distinct markers are labelled in mitochondria and cells for subsequent comparison. Step 2, isolated mitochondria are transferred by different AMT/T methods: **a** Coculture and noncontact co-culture (transwell). **b** Isolated mitochondria coincubated with cells. Methods "Magnetomitotransfer," "MitoCeption," "MitoPunch," and "centrifugation" methods are used to increase the efficiency of mitochondrial transfer. **c** Isolated mitochondria are injected in situ, intravenously (i.v.), or intranasally. **d** Autologous mitochondrial microinjection to improve oocyte quality and fertility outcome. Step 3, in vitro studies, quantitatively measure the efficiency of mitochondrial transfer by confocal microscopy, flow cytometry, or electron microscope. In the in vivo studies, subjects' behaviour and organ function are measured. Step4, the effect of mitochondrial transfer is evaluated. *MPF* mechanical plunger force, *CF* centrifugal force, *M plate* magnetic plate, *M beads* magnetic beads, *MF* magnetic force

Furthermore, due to the small amount of literature, inconsistencies in the detection methods used and detection times point of mitochondrial transfer efficiency, and other limitations (previous AMT/T experiments have lacked observations of more prolonged periods and generations of the posttransfer cells), it is difficult to compare the results. Therefore, detecting mitochondrial transfer efficiency and exogenous mitochondrial function requires a paradigm, and a comparison of existing methods is necessary. The comparison is summarized in Table1, and the flow diagram is shown in Fig. 2.

**Table 1:** A comparison of AMT/T methods

Methods	Donor	Recipient	Transfer efficiency	Therapeutic effect	Retention time	Refs.
Coculture	WJMSCs	143B ρ0 cells	NA	Proliferation↑ Aerobic viability↑ Cellular motility↑	45passages (135 days)	[83]
Coculture	MSCs	EMCs	12.4 ± 3.5% (24 h)	Necroptosis-like cell death of MSCs↑	NA	[84]
Coincubation	Mitochondria from MSCs	EMCs	48.9 ± 2.9% (24 h)	Necroptosis-like cell death↑	NA	[84]
Coincubation	Mitochondria from TAT‐dextran modified H9c2 cells	EMCs	170.3 ± 15.7% (24 h)	Bax to Bcl-2 ratio↓ Mitochondrial respiration↑	NA	[84]
Coincubation	Mitochondria from EMCs	EMCs	0.5–27.6% (mitochondria dose-dependent; 24 h)	Cellular viability↑ ATP production↑ Mitochondrial respiration↑	1 week	[85]
Coincubation	Mitochondria from MRC-5	MRC-5	0–17% (over 3 days)	Mitochondria respiration↑	NA	[63]
Magnetomito transfer	Mitochondria from MRC-5	MRC-5	78–92% (over 3 days)	Mitochondria respiration↑↑ Cell growth—	NA	[63]
MitoCeption	Mitochondria from MSC	MDA-MB-231 cancer cells	~ 1.9–7.1% (mitochondria dose-dependent; 24 h)	OXPHOS↑ ATP production↑ Cancer cell invasion and proliferation↑	NA	[62]
Adapted MitoCeption	Primary allogeneic mitochondrial mix	Peripheral blood mononuclear cells	5–40%; 60–100%; (mitochondria dose-dependent; cell type dependent; 1 h, 18 h)	Mitochondrial mass, function, and viability↑ P53↓ ROS↓ ATP production↑	NA	[86]
MitoCeption	Mitochondria from HEK293T cells	143BTK ρ0 cells/BJ ρ0 cells	~ 80–25% (2 h)	ATP production↑ Mitochondrial respiration↑	Stably retained in 143BTK ρ0 cells	[69]
MitoPunch	Mitochondria from HEK293T cells	143BTK ρ0 cells/BJ ρ0 cells	~ 50–10% (2 h)	ATP production↑↑ Mitochondrial respiration↑↑	Stably retained in both 143BTK ρ0 and BJ ρ0 cells	[69]
Photothermal nanoblade	Mitochondria from HEK293T cells	143BTK-ρ0 cells	~ 2%	Metabolism-related gene expression↑ Metabolic network activity↑ Metabolite levels↑	Stably retained	[67]
Cell fusion	Different donor	Different recipient	0.0001–0.5%	NA	NA	[67]

~ : Estimate; ↑: promote; ↓: reduce; —: no effect

*OXPHOS* oxidative phosphorylation, *MRC-5* human embryonic lung fibroblasts, *WJMSCs* Wharton’s jelly mesenchymal stem cells, *EMCs* endometrial gland‐derived mesenchymal cells, *NA* not available

### AMT/T in vivo

In addition to observed mitochondrial transfer in vitro experiments, mitochondria can also be injected directly into living organisms. The mitochondria used for injection can be autologous, allogeneic, or even xenogeneic. Doulamis et al. injected the allogeneic or autologous mitochondria of muscle cells into the IR-injured hearts of diabetic rats, both of which resulted in the recovery of left ventricular function and the reduction of infarct size [70]. Fu et al. isolated the mitochondria of HepG2 cells and then injected them into high-fat-diet-fed mice, effectively improving nonalcoholic fatty liver disease [71]. In addition, mitochondria can be injected directly into the damaged area or elsewhere. For example, Lin et al. injected mitochondria into the spleen to treat IR injured liver [72]. Additionally, researchers injected mitochondria directly into the regional ischaemic zone to repair myocardial damage more often in the past [73, 74]; recently, researchers have chosen to inject mitochondria into the left coronary ostium [75] or coronary artery [70]. Local intracerebral or systemic intra-arterial injection of mitochondria could significantly restore brain infarct area and neuronal cell death [76]. Moreover, intra-arterial injection or vascular delivery of mitochondria into the blood vessels were performed to treat acute kidney injury [67] or lung injury [68]. Currently, intravascular injections seem to be more prevalent and have led to better changes in blood biochemical indices and blood flow. Indeed, a recent study revealed that intact and functional mitochondria exist in human peripheral blood [77]. Moreover, there is much evidence that there are multiple mitochondrial components in the blood, such as cell-free circulating mtDNA [78], mitochondrial-derived vesicles [79], and mitochondrial-derived peptides [80], and these components are increased in the disease state. Although the significance of their presence in the blood and their relevance to disease is unclear, the presence of these components does demonstrate that mitochondria can play a signal-regulating role through the circulation to distant cells, even if fragmented. Accordingly, intravascular injection of mitochondria may be promising, but we need to understand the existence of mitochondria in the blood and the biological role of mitochondrial components in advance and fully consider the stability and whereabouts of mitochondria in the blood.

Human mitochondrial transplantation has been performed in the clinical setting. Emani et al. performed autologous mitochondrial transplantation. They isolated the mitochondria from patients’ rectus abdominis muscle and transplanted the mitochondrial into IR-injured hearts through epicardial injections. This autologous transplantation produces excellent results that ventricular function improved in all patients, and four out of five subjects successfully separated from ECMO support [81]. Moreover, Emani et al. introduced other potential optional methods for human hearts mitochondrial transplantation than the epicardial injection, such as transcoronary delivery, which is under investigation [81]. In clinical practice, the most mature technology is mitochondrial microinjection. Autologous mitochondrial microinjection can improve the quality of oocytes, help older women give birth, and reduce the incidence of disease in offspring. At present, autologous mitochondrial microinjection has been studied in humans, mice, pigs, cows, and other animals [82].

## Stem cell-derived mitochondrial transplantation

Stem cell-derived mitochondria transplantation refers to the transplantation of stem cells to damaged area and utilizes the spontaneous mitochondrial transfer of the stem cell to rescue the injured cells or the injection of stem cell isolated mitochondria into the injured area to repair the damage. Stem cells are the most primitive cells at the top of the origin of cell lines, and they have a high capacity for differentiation and self-renewal [87]. In addition, stem cells can differentiate into various tissues, organs, or functional cells of the human body; therefore, stem cells hold great promise for therapeutic tissue engineering and regenerative medicine [88]. Recent studies confirmed that stem cells could transfer mitochondria to adjacent cells, and the mitochondria provided by stem cells rescued adjacent cell respiration, induced cell reprogramming, and ultimately repair cells and increase cell function. It has been gradually recognized that mitochondrial transfer plays a critical role in injured tissue treatment by stem cells [9].

Among stem cells, MSCs are used chiefly to treat various diseases due to their unique advantages. First, in terms of technology, MSCs originating from the mesoderm have four advantages: (1) MSCs can retain their activity and regenerative ability after being stored at 80 °C; (2) the MSCs extraction approach is simple, and MSCs are easy to culture and cryopreserve; (3) MSCs rapidly replicate and have high potential for multilineage differentiation; and (4) immunoreactivity after MSC transplantation is minimal or even nonexistent (reviewed in [89]). In terms of mechanisms, MSCs have three significant characteristics: (1) MSCs have homing ability. After tissue injury, exogenous MSCs transplanted into animals preferentially home to inflammatory areas and injured tissues, while ischaemic injured tissue is more likely to attract the homing of MSCs [90]. Significantly, homing means that stem cells can be injected intravenously and carry their mitochondria for targeted transfer; (2) MSCs can differentiate into different tissue types to replace damaged cells. The differentiation tendency and proliferation ability of MSCs are influenced by different tissue sources [91]; (3) MSCs have paracrine functions by secreting soluble factors and releasing EVs to affect the surrounding cells and systemic pathophysiological processes, reducing inflammatory damage and promoting regeneration and repair [92]. The good paracrine characteristics of stem cells underlie the basis of their ability to be suitable mitochondrial donors and why they are more suitable for transplantation than other tissues.

Recent studies have shown that stem cells not only donate their normal mitochondria to damaged cells to resist oxidative stress, improve the metabolic state of cells but also take up and degrade mitochondria from damaged somatic cells, which promotes the proliferation of damaged cells and enhances their anti-apoptosis ability [84, 93]. The fate of mitochondria that degrade when they enter other cells has also been found in the nervous system, known as mitochondrial transcellular degradation [26, 94]. Mitochondrial transcellular degradation may be a more advanced and active manner of mitochondrial autophagy because it relieves the pressure on the recipient cell to degrade the mitochondria and helps the recipient cell better maintain mitochondrial homeostasis. In addition, transplanted stem cells deliver mitochondria and contain a large number of bioactive substances [95], which may work synergically with mitochondria.

Nevertheless, the risks of stem cell transplantation, such as tumorigenicity and potential immunogenicity, cannot be ignored. Multiple stem cell types have the property of inherent tumour tropism. Studies have shown that adipsin-derived stem cells promote the development of breast cancer cells by secreting adipsin and C-X-C ligand 1 (CXCL1) and CXCL8 [96, 97]. When neural stem cells (NSCs) and MSCs are injected through different routes into a mouse brain tumour model, NSCs and MSCs migrate to the tumor site [98, 99]. Beyond that, the surface of stem cells contains multitude surface antigens, including HLA-Class 1 antigens, which are not present on the mitochondrial membrane. Therefore, stem cells are much more immunogenic than isolated mitochondria. Moreover, in a recent in vitro study, it was found that mitochondria isolated from MSCs coincubated with EMCs had much higher mitochondrial transfer efficiency than MSCs coculture with EMCs [74].

In addition, further considerations are that mitochondria in stem cells are commonly peri-nuclear, showing spherical, fragmented and punctate shapes, and are condensed with underdeveloped cristae. It is generally regarded that the mitochondria in stem cells are in an immature state, exhibit lower levels of ROS, OXPHOS, and energy production [100]. If only treatment at the mitochondrial level is required for some diseases, mature mitochondria from skeletal muscle, heart muscle, the lungs, and the liver may be better theoretically [68]. However, because of safety and compatibility considerations, autologous mitochondrial transplantation is preferred in practice [32], while harvesting autologous mitochondria from somatic cells means causing secondary damage to the patient, whereas harvesting from stem cells does not. Second, the mature cells in those tissues cannot alleviate the constant demand for donor cells because of their limited in vitro expansion capabilities. Stem cells can be obtained from bone marrow, adipose tissue, dental pulp, and umbilical cord blood and can be expanded in large quantities in vitro to obtain sufficient mitochondria for treatment. Therefore, stem cell-derived mitochondria transplantation may be the best option for patients at this stage.

## The cellular and molecular mechanisms underlying mitochondrial transfer/transplantation

It is worth pointing out that only a portion of the mitochondria of cells conducts movement under physiological conditions. For example, only 10–20% of mitochondria in nerve cells progressively move under physiological conditions, while the majority of mitochondria remain stationary [11, 12]. However, under pathological conditions, certain signal stimuli such as oxidative stress, inflammation, and structural damage to cells trigger mitochondrial movement and mitochondrial transfer, which may help damaged cells restore their functions [13]. This section will introduce the specific signals that trigger mitochondrial transfer, the molecules involved in facilitating the mitochondrial transfer, and the cellular mechanisms of extracellular mitochondrial entry to clarify the underlying mechanism of mitochondrialr further.

### Triggers signals of intercellular mitochondrial transfer (Fig. 3)

**Fig. 3:**
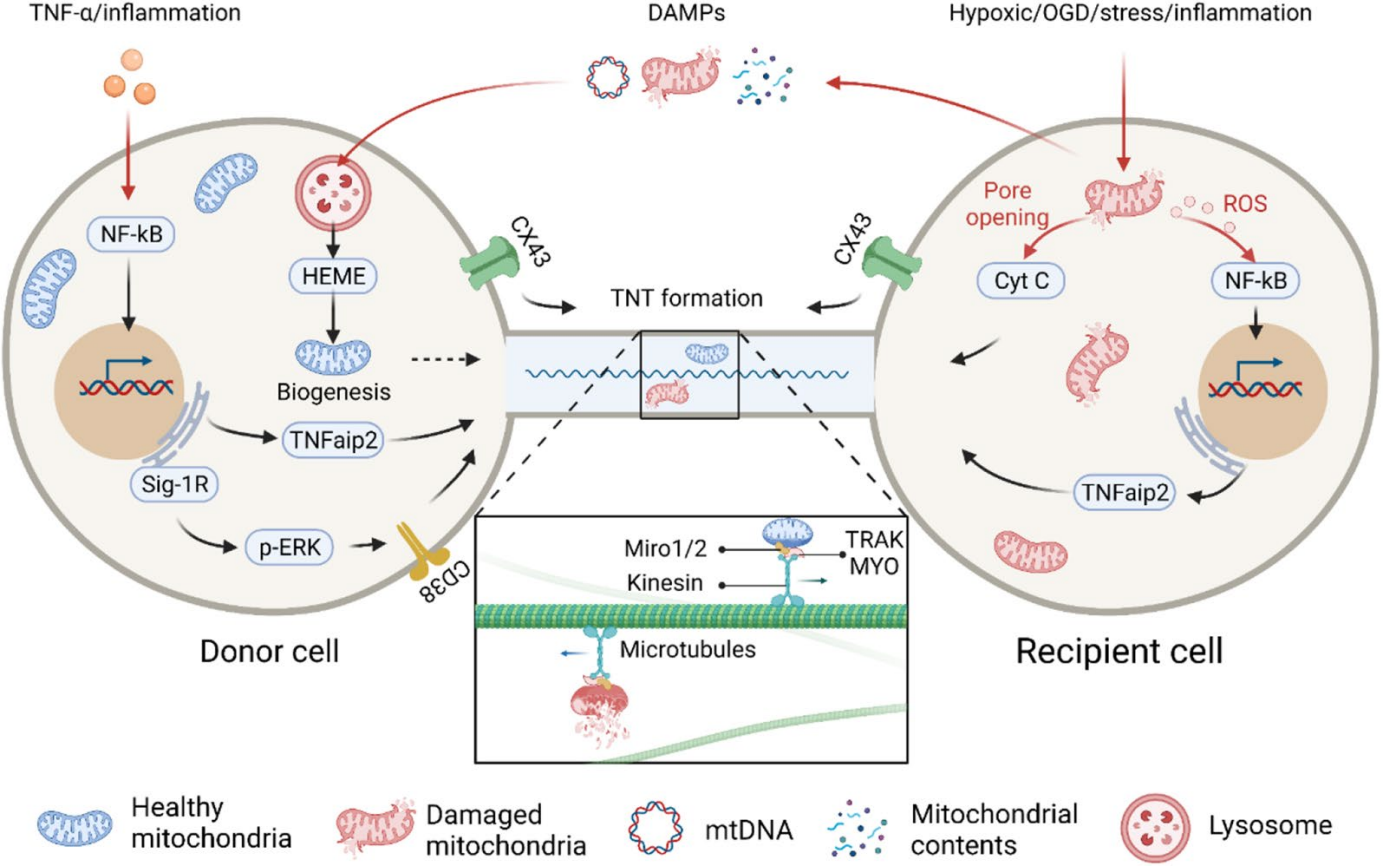
The mechanism of intercellular mitochondrial transfer. The red arrows indicate intercellular mitochondrial transfer trigger signals, and the black arrows indicate the signalling pathways involved in TNT formation and mitochondrial transfer

A prerequisite for mitochondrial transfer is that the cell can sense many different environmental signals and subsequently undergo foreign material absorption, trafficking, processing, and integration. Which signals trigger mitochondrial transfer is of great significance for further theory and treatment. Current evidence has proven that mitochondrial transfer between cells is often triggered by multiple intracellular and extracellular events of the recipient cell, such as hypoxic induction [101, 102] or OGD [20, 31, 103], drug-induced oxidative stress [21, 104], and inflammation [105, 106]. These events may act as “find me” or “rescue me” signals, recruiting appropriate donor cells to provide mitochondria to the recipient cells.

Most interestingly, damaged mitochondria in the recipient cells also act as “danger” signals that trigger mitochondrial transfer [93, 107, 108]. Both ROS and cytochrome C released by damaged mitochondria can promote mitochondrial transfer. For example, at the early stage of PC12 cell apoptosis, before caspase-3 was activated, cytochrome C released by the damaged mitochondria was shown to promote TNT formation. Moreover, although the results showed that caspase-3 was not associated with TNT formation, the treatment of cells with a pan-caspase inhibitor disturbed microtubule entry or assembly in TNTs [19]. In addition, Phinney et al. demonstrated that MSCs trafficked depolarized mitochondria to the plasma membrane by packaging mitochondria in vesicles in response to oxidative stress; the vesicles were then engulfed and reutilized by aggregating macrophages that enhance bioenergy [109]. Mahrouf-Yorgov et al. found that mitochondria released from damaged cells could act as DAMP signals and stimulate the expression of heme oxygenase-1 (HO-1) and the mitochondrial biogenesis of MSCs when they met and were absorbed, thereby enhancing the mitochondrial transfer and rescue ability of MSCs to damaged cells [93]. All of this evidence suggests that mitochondria may serve as an essential trigger signal that had previously been ignored.

### Molecular mechanism of intercellular mitochondrial transfer (Fig. 3)

TNFaip2/M-Sec is a widely expressed protein in mammalian cells. Membrane-associated M-Sec recruits active RalA, which elicits membrane deformation and facilitates TNT formation in cooperation with the exocyst complex and Lst1 [110]. The coculture of MSCs and corneal epithelial cells (CECs) indicated that the ROS produced by the pretreatment of CECs with rotenone could promote the expression of M-Sec by activating the transcription factor nuclear factor kappa B (NF-κB), thereby promoting the formation of TNTs in CECs and improving the efficiency of mitochondrial transfer from MSCs to CECs [104]. The proinflammatory factor TNF-α can also stimulate the expression of M-Sec by activating NF-κB in MSCs, thereby promoting the formation of TNTs and mitochondrial transfer between MSCs and injured cells [106].

Miro1 and Miro2 are Rho‐GTPases that are located in the mitochondrial outer membrane (MOM). They act as adaptor proteins to help mitochondria couple with microtubule motor proteins. Both Miros have two EH-hand Ca2+-binding domains and GTPase domains, and they are notably suitable for coordinating mitochondrial dynamics with cell signal regulation and local energy turnover [111]. In the current model of mitochondrial transfer, Miros on the MOM binds to the kinesin motor protein KIF5 directly [112] or with the help of accessory proteins such as TRAK1, TRAK2, MYO10 and MYO19 [113] to form a motor-adaptor molecular complex to facilitate and regulate mitochondrial movement along microtubules. Previous studies revealed that the overexpression of Miro1 rather than TRAK1 in MSCs could significantly increase the mitochondrial transfer efficiency and reverse rotenone-induced epithelial injury, while the knockdown of Miro1 in MSCs resulted in a loss of transfer efficiency and repair capacity [114]. The occurrence of mitochondrial transfer from mesenchymal multipotent stromal cells (MMSCs) to nerve cells was positively correlated with Miro1 expression in MMSCs [115]. The overexpression of Miro1 in MSCs showed a stronger ability to transfer mitochondria to nerve cells and promote injured nerve cell repair [116]. In addition, recent work revealed that knocking down or overexpressing Miro1 or Miro2 could affect the efficiency of mitochondrial transfer between nerve cells to almost the same extent [117].

**Fig. 4:**
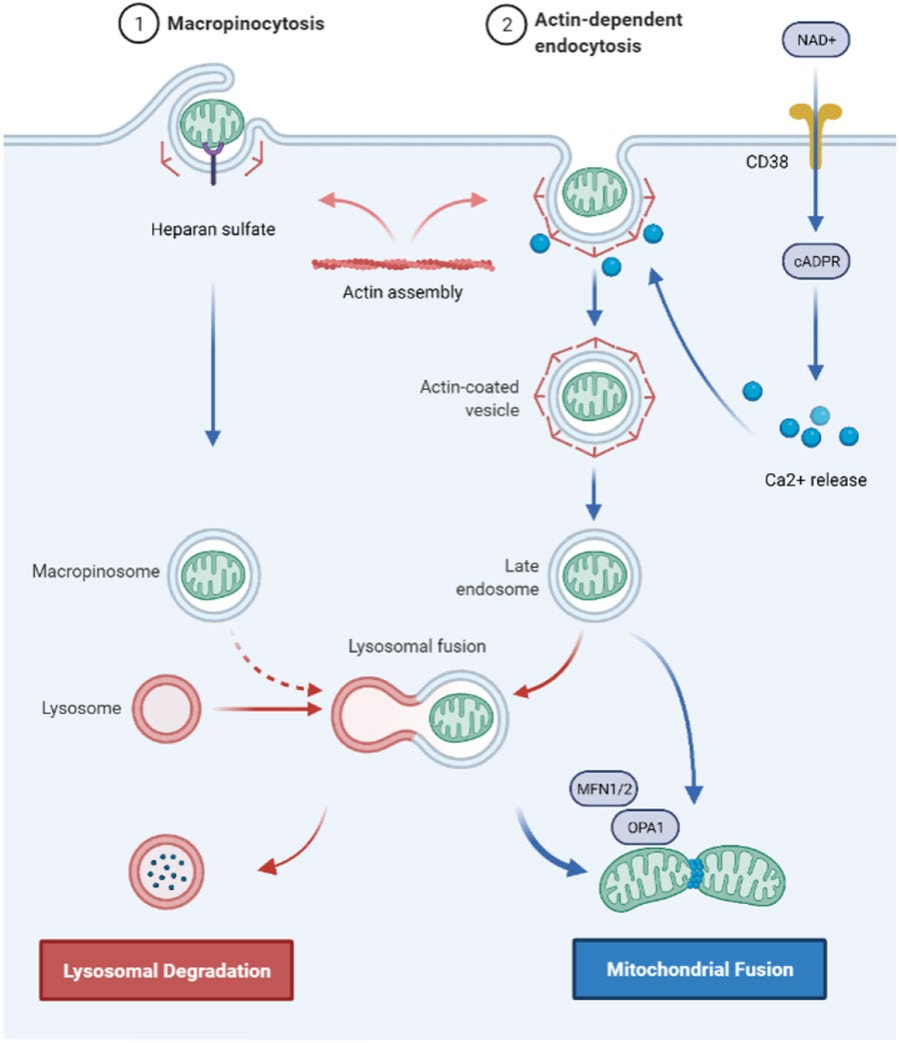
The putative mechanism by which cells engulf and internalize exogenous mitochondria. Exogenous mitochondria enter the cell via actin-dependent endocytosis or macropinocytosis and then form endosomes or macropinosomes. A small portion of exogenous mitochondria bind to lysosomes and are degraded; a large portion of exogenous mitochondria escape from endosomes, macropinosomes or lysosomes and fuse with endogenous mitochondria, which is mediated by MFN1/2 and OPA1

CD38 is a transmembrane glycoprotein that catalyses the synthesis and degradation of cyclic ADP-ribose (cADPR). In a study of cocultures of BMSCs and multiple myeloma cells, it was found that CD38 mediates the formation of TNTs, while inhibiting CD38 led to mitochondrial transfer reduction in-vitro and in-vivo [53]. In addition, the release of extracellular mitochondrial particles by astrocytes also required a calcium-ion-dependent mechanism mediated by CD38 and cADPR. The inhibition of CD38 reduced the extracellular mitochondria population and astrocyte-to-neuron mitochondrial transfer [26]. CD38-cADPR signalling was also involved in cell endocytosis. The researchers found that a large amount of NAD+ was released from human glioma cells through the cell membrane and bound to CD38 outside the cell membrane after starvation. Under the catalysis of CD38, NAD+ was converted into the messenger factor cADPR and re-entered the cell. Subsequently, cADPR acted on ER ryanodine receptors (RyRs) to initiate the release of Ca2+ from the calcium store. The released calcium ions rapidly accumulated inside the cell membrane and triggered cytoskeletal protein conformational changes. Cytoskeleton remodelling eventually led to cell membrane invagination via endocytosis to absorb extracellular mitochondria into the cell (Fig. 4) [118]. Furthermore, in vitro studies have shown that ER-resident transmembrane protein sigma-1 receptor (Sig-1R) enhances the expression of CD38 by activating ERK1/2, thereby promoting astrocyte mitochondrial transfer [119].

Sig-1R is located on the ER, and ER is a calcium ion storage site; this may indicate that ER mediates mitochondrial transfer as Sig-1R and calcium ion are all related to mitochondrial transfer events. Indeed, the ER is in tight proximity with mitochondria through the action of molecular tethers, which are called ER-mitochondrial contacts, and these contacts have been found to determine mitochondrial replication, division, and distribution [120, 121]. Recently, Gao et al. found that ER-mitochondrial contact mediated mitochondrial transfer between osteocytes [50]. The Mitofusin 2 (Mfn2), a GTPase that binds the ER to mitochondria, principally mediates the transfer [50]. Moreover, the CD38 stimulated ER-mitochondrial interaction may facilitate mitochondrial energy generation and redox homeostasis during mitochondrial transfer in astrocytes [122].

### The cellular mechanisms of extracellular mitochondrial entry (Fig. 4)

How extracellular mitochondria enter damaged cells needs more research, especially in vivo. It is widely recognized that cells absorb isolated mitochondria by endocytosis [118, 123], but there is disagreement about the type of endocytosis. Pacak et al. investigated the potential mechanisms by using inhibitors of clathrin-mediated endocytosis (methyl-b-cyclodextrin), actin mediated endocytosis (cytochalasin D), micropinocytosis[5-(N-ethyl-N-isopropyl)-amiloride], and TNTs (nocodazole) to treat cardiomyocytes and found that cardiomyocytes take up extracellular mitochondria through actin-dependent endocytosis [124]. Furthermore, Kesner et al. used macropinocytosis inhibitors [amiloride or 5-(N-ethyl-N-isopropyl)-amiloride] and clathrin inhibition-treatments (hypertonic sucrose, potassium depletion, and cytosolic acidification) to treat HepG2 cells and fibroblast cells and found that macropinocytosis inhibitors suppressed mitochondrial transformation in a dose-dependent manner, while the inhibition of clathrin-mediated endocytosis did not affect mitochondrial transformation [125].

Macropinocytosis is a type of pinocytosis in endocytosis that is actin dependent and forms large vacuoles; macropinocytosis is considered a pervasive and widely conserved process and may have participated in the first mitochondrial endosymbiosis event [126]. Macropinocytosis seems widely accepted since other studies have also demonstrated that MSCs and hepatocytes engulf isolated mitochondria mainly through macropinocytosis [85, 127]. Interestingly, macropinocytosis is known to be regulated by extracellular environmental signals [128]; this process does not require particles to interact with receptors on the cell membrane and is performed in all cells in an unspecific way [126]. However, the data of Kesner et al. showed that HepG2 cells and fibroblast cells selectively absorbed mitochondria that had intact outer membranes and that mitochondrial transformation required interaction with cellular heparan sulfate proteoglycan [125].

After the cell absorbs extracellular mitochondria, internalized mitochondria are transported to the endosome and lysosome. Cowan et al. showed that most exogenous mitochondria could escape from endosomes and lysosomal chambers and effectively fuse with the endogenous mitochondrial network of cardiomyocytes [123]. In addition, Mfn1, Mfn2, and optic atrophy 1 (OPA1) were found to facilitate the fusion of exogenous mitochondria with endogenous mitochondrial networks in cardiomyocytes and fibroblasts [88]. However, how mitochondria escape endosomes and lysosomes remains ambiguous. What factors influence the fate of mitochondria after entering cells, and which pathway affects the process of exogenous mitochondria integration with intracellular mitochondria? Additional studies are needed.

Notably, the cell types and culture conditions used in these experiments were different. The different results suggest that the way cells engulf and internalize mitochondria is not absolutely conserved and may be influenced by the environment. A summary of the cellular mechanisms of extracellular mitochondria entry characterized to date in mammalian cells is shown in Fig. 4.

## The therapeutic effects of mitochondrial transfer/transplantation on diseases

With mitochondrial transfer recognized and understood, researchers have begun to focus on the role that mitochondrial transfer plays in disease. The idea of using exogenous mitochondrial to treat disease was born. According to the existing literature, we discuss the therapeutic strategy that targets mitochondrial transfer/transplantation. In addition, we classify the diseases involved in the treatment of mitochondrial transfer/transplantation as mitochondrial-related diseases, such as metabolic disorders, ageing, tissue injury, and mitochondrial diseases caused by mtDNA deletion or mutations. Tumours and cancers are also discussed due to their importance and profound correlation with the mitochondrial transfer. Moreover, we also summarized mitochondrial transfer/transplantation therapy for diseases of different organs in Table 2.

**Table 2:** Summary of mitochondrial transfer/transplantation in different organ

Organ	Disease	Donor	Recipient	Methods	Therapeutic effect	Refs.
Brain	Sepsis-associated brain dysfunction	Pectoralis (allogeneic)	Mouse left lateral ventricle/BV2 microglia	Mitochondrial transplantation (i.c.v. injection)/mitochondrial coincubation	Cognitive impairment↓ Microglial polarization from the M1 phenotype to the M2 phenotype	[193]
Brain	Schizophrenia	Human lymphocytes/rat brain (xenogeneic)	Schizophrenia-derived lymphoblasts/rat prefrontal cortex neurons	Mitochondrial incubation/mitochondrial transplantation (DI)	Long-lasting cellular oxygen consumption and mitochondrial membrane potential↑ Attentional deficit↓	[194]
Brain	PD	HepG2 cells (xenogeneic)	Multiple tissues, including brain, liver, kidney, muscle, and heart tissues of mice	Mitochondrial transplantation (i.v. injection)	ETC activity↑ ROS level↓ Apoptosis and necrosis↓	[195]
Brain	PD	PC12 cells (allogeneic)/human osteosarcoma cybrids (xenogeneic)	PC12 cells/ rat dopaminergic neurons	Peptide-mediated mitochondrial incubation/peptide-mediated mitochondrial transplantation (i.c.v. injection)	Cell viability↑ Mitochondrial tolerance to 6-OHDA neurotoxicity↑ Motor function↑ Dopaminergic neuron deterioration↓	[143]
Brain	AD	HeLa cells (xenogeneic)	Brain and liver of mice	Mitochondrial transplantation (i.v. injection)	Cognitive performance↑ Neuronal loss↓ Gliosis↓ Mitochondrial dysfunction in brain↓ Mitochondrial activity in liver↑	[142]
Brain	IR injury	Pectoralis major (autologous)	Widespread distribution in the brain, mainly in ischaemic penumbra areas of the brains of rats	Mitochondrial transplantation (i.c.v. injection)	Cellular oxidative stress↓ Apoptosis↓ Astrogliosis↓ Neurogenesis↑ Brain infarct volume↓ Neurological deficits↓	[196]
Brain	IR injury	Rat MSCs (allogeneic)	Rat peri-infarct area of brain	MSC transplantation (intra-arterial injection)	Mitochondrial activity↑ Angiogenesis↑ Infarct volume↓ Functional recovery↑	[197]
Spine	Spinal cord injury	PC12 cells/soleus muscle (allogeneic)	Resident spinal cord cells of rats	Mitochondrial transplantation (DI)	Oxygen consumption rate↑ Long-lasting motor and sensory functions—	[198]
Heart	Myocardial infarction	hMADS (xenogeneic)	hMADS/endothelial or cardiac cells/mouse myocardium	Coculture/hMADS transplantation (surrounding the infarcted site injection)	HO-1 expression↑PGC1 -α expression↑ Plasma cardiac troponin t levels↓ Intracardiac caspase-3 protein expression↓	[93]
Heart	Anthracycline-induced cardiomyopathy	Human iPSC-MSCs (xenogeneic)	NMCs	Coculture (TNTs)/iPSC-MSC transplantation (intramyocardial injection)	Mitochondrial respiration↑ NMC viability↑	[106]
Heart	Anthracycline-induced cardiomyopathy	Human iPSC-MSCs/BM-MSCs (xenogeneic)	Mouse myocardium	iPSC-MSC/BM-MSC transplantation (intramyocardial injection)	Heart function↑ Myocardial damage↓ Myocardial fibrosis↓ Cell apoptosis↓ ATP production↑ Oxidative stress↓ Inflammation↓	[106]
Heart	IR injury	Pectoralis major of rabbit (autologous)	Rabbit myocardium	Mitochondrial transplantation (DI)	Creatine kinase MB↓ Cardiac troponin-I↓ Apoptosis↓ Infarct size↓ Oxygen consumption↑ ATP production↑ Precursor metabolites for energy↑ Cellular respiration↑	[74]
Heart	IR injury	Human adult cardiac fibroblasts (xenogeneic)/ rabbit liver (autologous)	Rabbit myocardium	Mitochondrial transplantation (DI)/mitochondrial coronary vasculature perfusion	End diastolic pressure↓ Positive dp/dt↑ Segmental shortening↑ Infarct size↓	[199]
Lung	Acute respiratory distress syndrome	Human MSCs (allogeneic)/murine alveolar macrophages (allogeneic)	Human monocyte-derived macrophages/mouse LPS-injured lung	Noncontact coculture (EVs)/instillation of alveolar macrophages treated with MSC-derived EVs (intranasally instilled)	Macrophages OXPHOS↑ Anti-inflammatory response↑ Phagocytosis↑ Lung injury↓	[132]
Lung	Acute lung injury	Mouse BMSCs (allogeneic)	Mouse alveolar epithelium cells	BMSC transplantation (airway-instilled)	ATP concentrations↑ Alveolar leucocytosis↓ Protein leakage↓ Surfactant secretion↑ Mouse survival↑	[30]
Lung	Airway injury and allergic airway inflammation	MSCs (allogeneic)	Lung bronchial epithelial cells of mice	MSC transplantation (intratracheal or intranasal route)	Caspase-3↓ Caspase-9↓ Apoptosis↓ Inflammation↓ Mitochondrial function↑	[114]
Lung	Alveolar epithelial-capillary barrier disruption	MSCs (allogeneic)	Primary human pulmonary microvascular endothelial cells/human small airway epithelial cells/lung	EV cocultured/EV transplantation (i.v. injection)	Barrier integrity↑ Mitochondrial respiration ↑ ATP turnover↑ Mitophagy↓ mtDNA replication↑ Inflammatory response↓ Mitochondrial function↑	[200]
Liver	Carbon tetrachloride-induced liver injury	Mouse liver (allogeneic)	Mouse hepatocytes	Mitochondrial co‐incubation/mitochondrial transplantation (i.v. injection)	Liver injury↓ Liver fibrosis↓ ROS level↓ ATP content↑ OXPHOS↑ Cell proliferation↓ Xenobiotic metabolism transformation↑ Protein homeostasis↑	[127]
Kidney	Diabetic nephropathy	Rat BM-MSCs (allogeneic)	Rat renal proximal tubular epithelial cells/mouse kidney	Mitochondrial transplantation (under the renal capsule of rats)/MSC transplantation (i.v. injected)/coculture/mitochondrial coincubation	Colony formation↑ Apoptosis↓ SOD2↑ Bcl-2↑ ROS production↓ Megalin and SGLT2 expression↑ Renal tubules structural restoration↑	[201]
Bone	Bone defect	Rat BMSCs (autologous/allogeneic)	BMSCs/parietal bone of rats	Mitochondrial coincubation /mitochondria-recipient BMSC transplantation (DI)	Cell proliferation and migration↑ Osteogenic differentiation↑ Bone defect repair↑ OXPHOS↑ ATP production↑	[148]
Bone	Osteoarthritis	Rat BMSCs (allogeneic)	Rat chondrocytes	Coculture	Mitochondrial membrane potential↑ Mitochondrial respiratory chain enzymes activity↑ ATP content↑ Apoptosis↓ Type II collagen secretion↑ Proteoglycan protein↑	[147]
Skeletal muscle	Skeletal muscle atrophy	Human umbilical cord- MSCs (xenogeneic)	Rat myoblast cells	Centrifugation	Cell proliferation↑ ATP content↑ MMP↑ PGC-1↑ AMPK/FoxO3/Atrogene pathway↓	[64]
Skeletal muscle	Tendinopathy	Human umbilical cord-MSCs (allogeneic)/rat L6 myoblasts (allogeneic)	Human tenocytes/rat Achilles tendon	Centrifugation/Mitochondrial transplantation (DI)	Tenomodulin↑ Collagen 1↑ MMP1↓ ROS↓ ATP production↑ Mitochondrial fusion↑ Mitochondrial fission↓ Apoptosis↓ Inflammation↓	[145]

↑: promote; ↓: reduce; —: no effect

*i.c.v. injection* intracerebroventricular injection, *hMADS* human multipotent adipose-derived stem cells, *NMCs* neonatal mouse cardiomyocytes

### Energy metabolism dysfunction

Mitochondria produce approximately 95% of cell's ATP, and when mitochondrial function is impaired, cellular metabolism to produce ATP cannot proceed normally, and the resulting chronic energy deficiency threatens cell survival and can even induces cell death to cause disease. For example, if insufficient ATP production in the axons of nerve cells results in the inability to generate action potentials, cognitive impairment, Parkinson’s disease (PD), or other diseases can occur [129]. The transfer/transplantation of mitochondria between cells is equivalent to installing new batteries in cells to supplement ATP production to support the survival of cells with impaired energy metabolism.

At present, many experiments have tested the effect of mitochondrial transfer on the bioenergetic levels of ATP in recipient cells in vitro. After 24 h of coculture of MSCs and BEAS-2B cells, MSCs could gradually transfer mitochondria to BEAS-2B cells through TNTs, attenuating ATP loss in BEAS-2B cells caused by cigarette smoke medium [130]. By coculturing WJMSCs with mtDNA-deficient ρ0 cells, WJMSCs were able to transfer their mitochondria to ρ0 cells and restore the expression of mitochondrial protein-encoding genes and the activity of the electron transfer chain (ETC) in the ρ0 cells, which helped the cells functional oxygen consumption and respiration. In addition, mitochondrial transfer helped ρ0 cells restore their cellular behaviour, including nonadherent proliferation, aerobic viability, and OXPHOS dependent cellular motility [83]. Moreover, ETC complex V-inhibitor-sensitive ATP generation and the metabolic shifting of ρ0 cells were restored [83].

The energy produced by the transferred mitochondria improves the function of various recipient tissues. The transplantation of isolated mitochondria into an ischaemic heart can reduce the infarcted area, increase the production of ATP, and improve cardiac systolic function [73, 74]. Furthermore, macrophages can increase their bioenergy through the phagocytosis of mitochondria in inflammatory and tissue injury situations, thus increasing their anti-inflammatory and phagocytic capacity [131, 132].

Diabetes mellitus is a metabolic disorder caused by insufficient insulin secretion by islet β-cells, and insulin secretion by islet β-cells mainly depends on the production of mitochondrial ATP stimulated by glucose. Under coculture conditions, human adipose MSCs could transfer mitochondria to human islet β-cells and enhance the bioenergy of damaged beta cells and thus their insulin secretion function [133]. Myocardial dysfunction, mitochondrial function, and cellular bioenergy were shown to be impaired in neonatal rat offspring exposed to pregestational diabetes mellitus and a high-fat diet. Transferring the mitochondria into the myocardium of these neonatal rat offspring in vitro could significantly enhance the respiration of the cardiomyocytes of male rats and reduce the apoptosis of the cardiomyocytes of male rats, but unfortunately, it increased the apoptosis of the cardiomyocytes of female rats [134]. Moreover, MSCs transferred mitochondria into fat-laden hepatocytes via TNTs under coculture conditions, and these transferred mitochondria may improve lipid load and tissue disorder in hepatocytes by providing oxidative capacity for hepatic lipid decomposition [135].

In addition to absorbing mitochondria to increase energy, cells can also maintain normal energy metabolism by expelling mitochondria. Emerging research shows that resident macrophages in the myocardium can absorb and degrade defective mitochondrial particles, which helps regulate myocardial homeostasis and prevent metabolic dysfunction in the heart [27]. More interestingly, macrophages in adipose tissue can take up mitochondria from nearby adipose cells. This adipose-macrophage mitochondrial transfer acts as a mechanism of immune-metabolic crosstalk and regulates metabolic homeostasis. However, this transfer is impaired in obese individuals due to heparan sulfate deficiency, leading to abnormal energy homeostasis and increased susceptibility to diet-induced obesity [136]. Therefore, the targeted regulation of intercellular mitochondrial transfer may be a new therapeutic strategy to improve metabolic diseases.

### Ageing-related diseases

Beyond being the centre of cell energy metabolism, mitochondria also convey signals and produce metabolites as regulators that affect all aspects of cell function and cause cellular senescence. It has been found without exception that with ageing, mitochondrial function in various tissues and organs of the organism decreases [137, 138]. Specifically, a reduction in the total number of mitochondria, mitochondrial membrane potential decline, and the accumulation of defective, senescent mitochondria have been shown [139, 140]. Whether mitochondria affect ageing, ageing affects mitochondria, or the two affect each other is unclear, but it is certain that good mitochondrial function is beneficial to the body. Current studies have shown that mitochondrial transfer/transplantation can enhance cell proliferation and mediate somatic reprogramming [48, 116]. Therefore, transferring healthy mitochondria into senescent cells may become a new method to reverse ageing.

The ageing of the skin is divided into chronological ageing caused by age and photoaging caused by light. Ultraviolet radiation (UVR) is the main factor leading to premature skin ageing, and may also lead to skin cancer [141]. It has been reported that a simplified MitoCeption method ‘primary allogeneic mitochondrial mix’ can significantly reduce the expression of P53 under UVR and repair UVR-damaged cells by restoring metabolic activity, mitochondrial quality, and the loss of mtDNA sequence stability [86].

Neurodegenerative diseases associated with ageing are also common and involve dysfunction of mitochondria. A study showed that i.v. injection of intact functional mitochondria in Alzheimer’s disease (AD) mice significantly reduced neuronal loss and gliosis in the hippocampus and significantly increased the activities of citrate synthase and cytochrome c oxidase, which reached the activity levels of non-AD mice, and the cognitive performance was considerably better than that of AD mice without mitochondrial injection [142]. Mitochondria were injected into the medial forebrain bundle of PD rats, which confirmed that mitochondrial transplantation could improve the mitochondrial function of substantia nigra neurons. Three months after transplantation, it was found that the activation function of PD rats was also improved, accompanied by a reduction in the loss of dopaminergic neurons in the substantia nigra dense region [143].

In addition, by simple centrifugation, intact mitochondria can be transferred to dexamethasone-treated atrophic muscle cells, blocking the AMPK/FoxO3/Atrogene pathway that causes muscle atrophy [64]. After injecting MSCs or MSCs pretreated with isolated mitochondria into the muscle of dystrophic α-sarcoglycan-deficient mice, the process of angiogenesis and myogenesis was improved [144]. The transfering mitochondria into damaged tenocytes or rat models of tendinopathy significantly attenuated inflammation and apoptosis, restoring collagen production, which may be due to enhance mitochondrial fusion [145]. Sarcopenia is one of the most striking effects of ageing, and mitochondria are the organelles responsible for the progression of sarcopenia as they are vital regulators of various factors that lead to the aetiology of the condition, such as ATP production, ROS, proteostasis, and apoptosis, as well as inflammation and Ca2+ handling [146]. The transfer of healthy mitochondria to skeletal muscle to maintain skeletal muscle health in elderly individuals will be an exciting subject of future research.

Accumulative evidence suggests that mitochondrial transfer can reduce chondrogenic stress and positively affect bone [7, 50, 147]. However, with increasing age, the Mfn2 protein that mediates mitochondrial transfer between osteocytes decreases, leading to impaired mitochondrial distribution and transfer in the dendritic network of osteocytes [50]. Therefore, the regulation of mitochondrial transfer may have positive implications for maintaining osteocyte homeostasis during ageing. A recent study showed that autologous mitochondrial coincubation with BMSCs enhanced the proliferation, osteogenesis, and migration of BMSCs, and OXPHOS activity and mitochondrial ATP production in BMSCs were increased after mitochondrial acquisition [148]. Moreover, transplanted BMSCs which transferred mitochondria into rat cranial bone defect sites could promote bone defect healing more efficiently than no treatment [148]. As a result, mitochondrial transfer/transplantation during the ageing process may have a positive significance in fighting nervous system diseases and musculoskeletal system diseases that have affected elderly individuals for a long time.

### Tissue injury

Mitochondria are essential organelles for cell survival. However, in some tissue injuries, such as IR injury, the disorder of mitochondrial structure and function may be an actual cause of the injury, especially in the energy-intensive organs of the body [149]. When cells cannot obtain enough oxygen due to tissue ischaemia, the energy metabolism of cells changes to glycolysis and produces a large number of acidic products, which induces cellular acidosis and damages subcellular structures. Ironically, with the prolongation of anoxia, ATP is further hydrolysed. Mitochondria cannot effectively synthesize ATP but need to consume ATP to maintain membrane potential stability and eventually become energy consumers of cellular ATP. Furthermore, ATP hydrolysis will reduce intracellular Ca2+-ATPase and Mg2+-ATP activity, leading to calcium overload; when reperfusion occurs, the mitochondria "instinctively" absorb Ca2+, which in turn causes the mitochondrial permeability transition pore channel to open, leading to cell death. Therefore, in addition to the timely supply of oxygen and energy, it is imperative to find suitable and healthy mitochondria to replace mitochondrial function and improve cellular respiration at the ischaemic injury site.

The McCully group investigated the therapeutic effects of mitochondrial transfer/transplantation at different time points in IR injury. Their data showed that the myocardial infarct caused by IR could be ameliorated by injecting the mitochondria-containing vehicle before ischaemia or two hours after perfusion [75, 150]. Furthermore, they have made incremental progress in the technique of mitochondrial transplantation. For example, they have explored the therapeutic effect of mitochondria from different muscle sources on cardiomyocytes in culture, and they have used autologous muscle mitochondria to treat heart failure and cardiac ischaemia in humans and animals [81, 151, 152]. Moreover, it is worth mentioning that they showed that donor mitochondrial isolated from different myofiber types and cardiac muscle are equally effective for recipient hypertrophied cardiomyocytes [151]. In turn, they also treated acute limb ischaemia by injecting cardiac mitochondria into the injured limb muscle [153]. Such methods are feasible theoretically and practically since cardiac and skeletal muscle show numerous physiological properties in common and both originate from the mesodermal lineage. Furthermore, in vitro studies have shown that recipient cells that harvest the mitochondria from the heart, lungs, and muscle, which have higher energy requirements, display a more robust respiratory profile than recipient cells that harvest the mitochondria from the spleen or kidneys, which have lower energy requirements [68]—suggesting that such high-energy-demand organs are theoretically suitable mitochondrial donors.

To explore the diverse methods of mitochondrial delivery, the McCully team experimented with vascular injection and in situ injection. They found that intracoronary delivery of mitochondria was faster and mitochondria were more widely distributed in the myocardium than direct myocardial injection of mitochondria in the treatment of myocardial injury. In contrast, direct injection (DI) could obtain a higher concentration of mitochondria in the target area [149]. They also performed mitochondrial transplantations via pulmonary artery vascular delivery or tracheal aerosol delivery, which improved lung mechanics and reduced lung tissue damage [154], and they performed mitochondrial transplantations via renal arteries, which protected against renal IR injury [155]. Therefore, mitochondrial injection can be conducted directly in situ or near blood vessels, which both achieve an excellent saving effect, depending on the specific type of organ and the difficulty of the operation.

In addition to isolated mitochondrial transplantations, stem cell-derived mitochondrial transplantation can also play a positive role in treating IR injury. The destruction of mitochondrial structure accompanies cell/tissue IR injury, releasing ROS, ATP, mitochondria, or incomplete mitochondrial components such as TFAM proteins or mtDNA into the extracellular milieu, which act as DAMPs, leading to the worsening of tissue injury and the inflammatory response. Studies have shown that MSCs can sense the extracellular mitochondria released by damaged cells and initiate the exchange of mitochondria with damaged cells to save the cell [93]. In vitro studies have shown that cocultured MMSCs can effectively transfer mitochondria to astrocytes when they are exposed to the ischaemic damage associated with ROS elevations. Intravenous MMSCs have also been shown to increase the number of astrocyte mitochondria and improve neurological recovery in animals that have suffered a stroke [116]. Therefore, implanting stem cells into damaged tissue and using stem cell-derived mitochondrial transplantation to promote recovery may also be feasible treatments. From a stem cell perspective, in addition to transferring mitochondria, EVs released by stem cells can also facilitate mitochondrial antioxidant defence and enhance ATP generation by activating the Keap1-Nrf2 signalling pathway of recipient cells, which protects cells from oxidative damage by reducing mitochondrial fragmentation, normalizing mitochondrial membrane potential, and increasing mtDNA copy number [156]. However, at present, much of the evidence of stem cell-derived mitochondrial transplantation for the treatment of IR injury has come from in vitro experiments, and more in vivo experimental research is needed.

### Diseases associated with mtDNA mutations and deletions

The mtDNA is not protected by histones and is highly vulnerable to oxidative damage. Damaged mtDNA lacks an effective DNA repair mechanism. In addition, mtDNA continuously synthesized throughout the cell cycle, and the DNA polymerase responsible for replicating mtDNA has poor proofreading ability; therefore, mtDNA is prone to mutations and deletions [157]. However, mtDNA sequences have no introns except for a small segment of the D-ring region; therefore, mitochondria are highly susceptible to mtDNA mutations and deletions.

At present, there are two theories. One is that only when the mutant mtDNA reaches the mutation threshold will it cause mitochondrial dysfunction; that is, the pathogenicity of mtDNA mutation depends on the ratio of mutant to wild-type mtDNA [158]. Another theory is that the absolute level of wild-type mtDNA, rather than the ratio of mutant to wild-type mtDNA, is an essential determinant of whether heterogeneously pathogenic mtDNA mutations cause mitochondrial dysfunction or disease [159]. Currently, total mtDNA copy number loss is associated with PD, embryo viability, and familial amyloid polyneuropathy and may serve as a biomarker of diseases [160–162]. In contrast, a study of blood samples from 14,176 people, including patients with reduced kidney function and healthy individuals, showed that the higher the mitochondrial copy number, the lower the risk of metabolic syndrome and type 2 diabetes [163]. Increasing mtDNA copy number improved arterial mitochondrial respiratory function and delayed vascular ageing in mice [164]. Likewise, Jiang et al. showed that heterotypic mtDNA mutagenesis-induced male infertility could be saved by increasing the total number of mtDNA copies without changing the mtDNA mutagenesis load [165]. Considering that there are no good tools for the targeted clearance of heterogeneous mitochondria and mitochondrial gene-editing technology is rudimentary, manipulating mtDNA total copy numbers may be an effective strategy to treat heterogeneous pathogenic mtDNA mutation diseases in the future. Coincidentally, AMT/T technology can increase mtDNA copy number and reduce the mutation ratio while avoiding mtDNA editing, which brings about the possibility of treating mtDNA mutations and deletions.

The same mtDNA site mutation can lead to different disease phenotypes. For example, mtDNA A3243G mutation can lead to mitochondrial encephalopathy lactic acidosis and stroke-like episodes (MELAS) or chronic progressive external ophthalmoplegia (CPEO), and maternally inherited diabetes and deafness (MIDD). In addition, the same disease phenotype can be caused by mutations of different mtDNA sites, such as MELAS, myoclonus epilepsy associated with ragged-red fibres (MERRF), and Leber’s hereditary optic neuropathy (LHON). Therefore, mitochondrial transfer/transplantation may be suitable for treating diverse mitochondrial diseases caused by mtDNA mutations or deletions.

Under no-contact coculture conditions, WJMSCs were found to transfer healthy mitochondria via F-actin structure to MERRF cybrid cells with a high mtDNA A8344G mutation rate. This transfer partially reduced the A8344G mutation load but was sufficient to reverse the ROS expression and oxidative damage induced by the A8344G mutation and improve the bioenergetics [166]. Similarly, Lin et al. found that WJMSCs could also transfer healthy mitochondria via TNTs to rotenone-stressed MELAS fibroblasts under no-contact coculture conditions, thereby eliminating the A3243G mutation burden to an undetected level, saving mitochondrial function and reducing apoptosis [167]. Furthermore, WJMSCs could transfer mitochondria into ρ0 cells under coculture, thereby restoring the attachment-free proliferation, aerobic viability, and OXPHOS-dependent cellular motility of ρ0 cells [83]. Nevertheless, the data of Young Min Cho et al. showed that MSCs did not transfer their mitochondria to hybrid cells containing the A3243G or 4977 bp deletion but transferred their mitochondrial only to ρ0 cells under coculture condition [10]. Although ρ0 cells are considered to be the well-characterized model of extreme mitochondrial deletion, there are no cells in healthy individuals or patients with mitochondrial diseases that are entirely free of mtDNA other than red blood cells. Therefore, it is imperative to focus on mtDNA-containing ρ+ cells and use more efficient methods to transfer mitochondria into ρ+ cells. Multiple AMT/T experiments have been successfully proven to enhance the transfer efficiency of mitochondria to recipient cells, either ρ0 cells or ρ+ cells, thus restoring cellular respiration and improving cell viability [61, 64, 66]. For example, the peptide-mediated delivery research showed that the mitochondrial transfer efficiency to ρ+ MERRF cybrid cells and ρ0 cells could reach 77.48% and 82.96%, respectively [61]. In addition, Teitell et al. transferred chloramphenicol-resistant mitochondria to chloramphenicol-sensitive ρ+ MELAS hybrid cells using Mitopunch. Then selection in chloramphenicol-supplemented media showed that chloramphenicol-resistant mtDNA had entirely and permanently replaced the endogenous mutant mtDNA [66].

Mitochondrial transfer/transplantation is now known to play critical roles in mitochondrial function and metabolic rescue and is considered to offer great promise for treating mitochondrial diseases [168]. However, most of the available information on mitochondrial transfer to treat mtDNA mutations and deletions is based on in vitro experiments. Supporting experimental evidence from animal studies and individual clinical case reports is lacking. Patients with mitochondrial deletion diseases, such as patients with LHON, are primarily homoplasmic, i.e., they carry only mutated mtDNA [169]. In this case, the transplantation of healthy mitochondria may increase mitochondrial heterogeneity, while autologous mitochondrial transplantation would mean the transfer defective mitochondria. Moreover, it may be challenging to remedy a systemic mtDNA copy number reduction by mitochondrial transfer/transplantation, the amount of mitochondria provided is also a significant challenge.

### Cancer

In 1920, Otto Warburg observed that hepatocellular carcinoma cells preferentially use glycolysis to produce ATP, in contrast to normal cells, which use OXPHOS of under aerobic conditions. He proposed mitochondrial respiratory defects as a potential basis for aerobic glycolysis and cancer, known as the “Warburg effect”. In addition, previous studies have found changes in the morphology and dynamics of mitochondria in various tumour cells [170]. Specifically, mtDNA abnormalities, including mtDNA mutations, mtDNA copy number abnormalities, mtDNA deletion and mtDNA microsatellite instability, have been observed throughout cancer development [171, 172]. Therefore, mitochondrial abnormalities have also been considered to be involved in the formation and progression of tumours. Treatments targeting tumour mitochondria have been developed, and the transfer of healthy mitochondria into tumour cells may be a good idea.

Nevertheless, we should be aware of the following: (1) Not every tumour cell shows the Warburg effect; some tumour cells show the Warburg effect and normal mitochondrial function, and some tumor growth even showed dependence on mitochondrial OXPHOS [173]; (2) mitochondria correlate with the occurrence and development of tumours. However, their relationship cannot be explained in a unified and straightforward way due to tumour heterogeneity; (3) mitochondria are likely to be 'hijacked' and exploited by tumor cells, which reprogram mitochondria and strictly control mitochondrial quality through autophagy and biogenesis to help tumorigenesis and development [174, 175]; and (4) from the perspective of mitochondrial transfer/transplantation, the essence of mitochondrial transfer/transplantation is to increase the healthy mitochondrial quality of the recipient cells, thereby improving the survival of the recipient cells. Therefore, mitochondrial transfer may enhance tumour viability. In vitro and in vivo studies have shown that increasing mtDNA copy numbers effectively promotes microsatellite-stabilized colorectal cancer cell survival and metastasis [171]. In contrast, Bonekamp et al. identified specific mitochondrial transcription inhibitors that can suppress tumour growth by destroying mtDNA transcription [176]. Therefore, increasing the number of healthy mitochondria in tumour cells does not necessarily mean improved cancer survival.

Converging evidence from different laboratories has shown spontaneous mitochondrial transfer between tumours and other cells, the pattern of which is mainly through TNTs and depends on the TME [55, 177]. Lu et al. detected spontaneous mitochondrial transfer between heterogeneous bladder cancer T24 cells and RT4 cells via TNTs, which increased the invasiveness of RT4 cells [178]. Pinto et al. found that TNTs helped glioblastoma (GBM) stem cells transfer mitochondria in tumour organoids and participated in the formation of tumor networking with tumour microtubes, which may contribute to tumour progression and therapeutic resistance [35]. Interestingly, GBM stem cells and PC12 cells showed increased TNT connections or increased microtubules in TNTs when exposed to X-ray or UV light, respectively, and both showed the upregulation of mitochondrial transfer, suggesting that TNT-mediated mitochondrial transfer has a protective effect on tumour cells [19, 35]. In addition to TNTs, tumour cells also transfer mitochondria by EVs. With electron microscopy, Salaud et al. observed that tumour-activated stromal cells, which are crucial components of the TME in glioblastoma, could transfer mitochondria to primary GBM through both TNTs and EVs [179]. Moreover, mtDNA and other components of mitochondria have been found in the circulating EVs and exosomes of cancer patients. Although the mitochondria in EVs are not complete, their packaging, release, uptake, and incorporation mode are also not transparent, but the mitochondrial components contained in EVs inevitably affect cancer progression [180, 181].

The researchers also established tumour-derived ρ0 cells to model the phenotype of tumour with extreme mtDNA damage. Interestingly, B16 ρ0 and 4T1 ρ0 cells (tumour cells with mtDNA deletion) showed delayed tumour growth. Nevertheless, when B16 ρ0 and 4T1 ρ0 cells were injected into mice, the tumour cells harvested mtDNA from the host mouse and thus restored their respiratory function and tumorigenic potential [182]. Moreover, the knockdown of essential mitochondrial complexes I and II also significantly inhibited tumorigenicity of B16 and B16 ρ0 cells, and B16 ρ0 cells were found not to form tumours unless mtDNA was obtained. When B16 ρ0 cells were transplanted into mice or cocultured with MSCs isolated from mice, the B16 ρ0 cells absorbed the entire mitochondria of the host to restore respiration and tumorigenesis [11]. Likewise, GBM ρ0 cells continued to proliferate after the clearance of mitochondria but more slowly than the GBM ρ+ cells [179]. The mitochondria isolated from MSCs were incubated with GBM ρ0 and GBM ρ+ cells and GBM ρ0 cells took up isolated mitochondria within 24 h of incubation and formed a mitochondrial network by 72 h [179]. However, the FACS data and a derived mathematical model showed that GBM ρ+ cells took up the mitochondria more rapidly than GBM ρ0 cells [179], in contrast to the uptake ability of normal cells-derived ρ0 and ρ+ cells observed in previous studies [10]. A549 ρ0 cells, whose parental cell lines were lung adenocarcinoma cell lines and could not proliferate due to lack of mitochondria, could obtain mitochondria when coculture with human BMSCs or human skin fibroblast cells to help them restore their proliferation ability [183]. Ippolito et al. found that cancer-associated fibroblasts (CAFs) contributed to prostate cancer (PCA) malignancy through mitochondrial transfer [184]. Furthermore, they pointed out that few PCA cells could absorb the exogenous mitochondria supplied by CAFs in the absence of CAFs, while pretreatment with CAF-conditioned medium could significantly enhance the ability of PCA to receive mitochondria. This phenomenon may be because the CM of CAFs contains cytokines released by CAFs that can simulate the TME in vivo, especially the lactic acid produced by CAFs, which may play a significant role in changing the demands of PCA cells on mitochondria [184, 185]. These experiments provided mitochondria-deficient tumour cells with the TME cells within the TME, and the data showed that the TME is critical to the ability of a tumour to acquire mitochondria and thus restore respiration and promote tumorigenesis.

Mitochondrial transfer between tumour cells or between tumour and the TME has also been shown to promote drug resistance. Mitochondrial transfer of T-cell acute lymphoblastic leukaemia (T-ALL) to MSCs via TNTs reduced oxidative stress under the action of chemotherapeutic drugs, while the inhibition of mitochondrial transfer by cytochalasin D treatment significantly reduces drug resistance of T-ALL cells [21]. With ROS-inducing chemotherapy, MSCs can become CAFs and rescue B-ALL cells by transferring mitochondria via TNTs. Mitochondrial depletion in MSCs or interference with TNT formation through microtubule inhibitors can reduce the transfer of mitochondria from MSCs to B-ALL, thus preventing the rescue function of MSCs [186]. Pasquier et al. found that TNT-mediated mitochondrial transfer occured between breast cancer cells and MSCs or endothelial cells (ECs), but occurs preferentially between ECs and breast cancer cells, and breast cancer cells that obtained EC mitochondria exhibited chemoresistance [187]. In conclusion, the TME and its interaction with other cells should be fully considered in tumour cell mitochondrial transfer research, which is the key to preventing tumour cells from benefiting from mitochondrial transfer.

Nevertheless, with the elucidation of the mechanisms underlying mitochondrial transfer in tumours, researchers have presented new mitochondrial transfer/transplantation therapies to treat tumours. Díaz-Carballo et al. found that in chemotherapy-resistant GBM, mitochondria were associated with syncytin-1/2 transfer between cancer cells via TNTs or were absorbed across the cancer cell membrane. Inhibiting syncytin-1/2 by their antibodies almost completely suppressed mitochondrial uptake via the cancer cell membrane, reducing tumour drug resistance [188]. In addition, data from Yu et al. suggested that mitochondrial transfer suppressed melanoma cell proliferation in vitro via cell cycle arrest and increased cell apoptosis [189]. Chang et al. demonstrated that through mitochondrial coincubation or pep-1-mediated mitochondrial delivery, the progression of MCF-7 breast cancer cells was significantly inhibited; these MCF-7 breast cancer cells showed an increase in cell apoptosis and a decrease in cell proliferation. Moreover, mitochondrial transplantation significantly reduced doxorubicin and paclitaxel resistance in both the MCF-7 and MDA-MB-231 cell lines [190]. Sun et al. found that the transfer of healthy mitochondria mediated by the coincubation of starved human glioma (U87) cells with healthy mitochondria stimulated the expression of tricarboxylic acid cycle-related genes and proteins, increased aerobic respiration, weakened glycolysis, reactivated the mitochondrial apoptotic pathway, and suppressed the malignant proliferation of U87 cells [118]. Whereas, the transfer of defective mitochondria carrying the A8344G mutation did not inhibit breast cancer cell proliferation but instead promoted it [190].

Moreover, Roushandeh et al. found that the transplantation of healthy fibroblast-derived mitochondria into HeLa ρ0 and Sas ρ0 cells restored the proliferation of these cancer cells in conventional media but increased their susceptibility to cisplatin-induced apoptotic death [191]. Cisplatin is a standard cancer treatment in humans. However, cisplatin has side effects such, as cognitive impairment. Alexander et al. nasally delivered human MSC-derived mitochondria to cisplatin-treated mice and found that these exogenous mitochondria could enter the brain meninges and parenchyma and be rapidly internalized by cells, restoring brain structure and function. Some delivered mitochondria even reached the hippocampus and altered the hippocampal transcriptome [192]. Therefore, mitochondrial transplantations may also improve the side effects of chemotherapy for tumors, but ensuring that mitochondria are transferred to target cells while not being taken up and utilized by tumour cells is also an intractable clinical problem.

## Risk and challenges

There is extensive literature describing mitochondrial transfer/transplantation, introducing novel techniques and methods regarding mitochondrial transplantation. However, some works of literature have been called into question and disputed in some detail, such as the validity of transplantation, the methods of mitochondrial administration and exogenous mitochondrial compatibility. Despite the outstanding safety characteristics and excellent in vivo efficacy and clinical case studies, doubts remain regarding mitochondrial transfer/transplantation safety in humans, as its side effect is not fully described. This section discusses the possible risks and challenges of mitochondrial transfer to achieve safe and widespread medical applications.

### Advantages of mitochondrial transfer/transplantation

Growing evidence has made targeted mitochondrial transfer a promising potential treatment for a variety of diseases, and mitochondrial transfer is receiving increased attention. In recent years, mitochondrial-targeting theories and agents such as NMN, MitoQ, SS31, CoQ10, CsA, and several small-molecule agents for targeting mitochondria have been proposed. However, the development and application of mitochondrial-targeted drug therapy are still slow, and the development of mtDNA editing technology is significantly behind that of nuclear DNA editing technology. Hampered by the conundrum of transporting RNAs into mitochondria, current gene modifying approaches cannot work effectively inside mitochondria [202]. Mitochondrial transfer opens a new door by providing additional opportunities to treat diseases. The treatment modality of transferring entire healthy mitochondria to recipient cells is simple and straightforward but has profound therapeutic implications.

Of course, mitochondrial transfer/transplantation as a treatment is in its infancy and has many blind spots. Spontaneous mitochondrial transfer undoubtedly helps maintain tissue homeostasis and revitalization, but its occurrence is rare, and not every tissue and organ shows strong mitochondrial transferability. Enhancing or suppressing spontaneous mitochondrial transfer to treat diseases may be less desirable and dangerous. For example, excessive GJC or HC opening may affect cellular homeostasis and induce cell death [29, 203]. TNTs are involved in diseases, especially as channels for the propagation of infectious bacteria and viruses between cells and tumour cell invasion and metastasis [16]. Studies on inducers and inhibitors of TNTs are immature since little is known about their underlying formation from the perspective of which specific molecules are involved; their biogenesis, fusion, and maturation for cargo transport are undetermined, and no comprehensive TNT inducers have been discovered at present [16, 204]. Thus, additional research is required to elucidate the molecular mechanisms underlying spontaneous mitochondrial transfer. AMT/T seems superior and more universal, transferring mitochondria into recipient cells with higher efficiency [32]. Current disease treatments also use AMT/T rather than enhancing spontaneous mitochondrial transfer.

In addition, stem cell-derived mitochondria transplantation is promising for mitochondrial transfer applications. It has two advantages: one is that it can secrete or receive cytokines to stimulate more efficient mitochondrial transfer and material exchange between donor and recipient cells; the other is that the damaged mitochondria in recipient cells can be absorbed and degraded by stem cells [93].

For now, stem cell-mediated mitochondrial transfer is one of the surprising serendipitous findings in the field of stem cell transplantation and is considered a novel effect mechanism for stem cell therapeutic efficacy. Regrettably, in regard to specific diseases, there should be specific mitochondrial transfer treatment options, but studies comparing the effectiveness of AMT/T and stem-cell-derived mitochondrial transplantation in specific disease conditions are lacking.

### Methods of mitochondrial administration

Current studies all point out that a single administration of mitochondria has a therapeutic effect that is not long-lasting; therefore, repeated administration for prolonged periods is needed. Additionally, the time point of administration, the cycles of administration, and the number of doses need further experimental research and rigorous clinical investigation (some of the subjects addressed in the review [205]). Furthermore, whether mitochondrial transplantation can be combined with other drugs is also worth considering. For example, a transactivator of transcription dextran complexes significantly enhanced the uptake of exogenous mitochondria by cells and improved the protective effect of mitochondria on oxidative stress in neonatal rat cardiomyocytes [84]. Treatment with the ALDH2 activator ALDA-1 significantly improved the cardiomyocyte oxygen consumption rate and basic mechanical function induced by mitochondrial transplantation [206].

As described above, mitochondria can be administered in situ, in the tail vein, or intranasally. However, as reports of in vivo studies have increased, questions have been raised about the viability of mitochondria at high extracellular calcium levels [207]. There is a substantial potential difference between the two sides of the inner mitochondrial membrane, usually between 150 and 180 mV (negative inside), which can reach 180–220 mV in the most extreme cases. The mitochondrial potential difference is far more significant than that of any other organelle (plasma membrane potential, 30–60 mV, negative inside), which means that mitochondria are naturally more capable of attracting cations [208]. When isolated mitochondria were in a calcium-containing medium, mitochondria rapidly took in surrounding calcium, leading to the irreversible opening of the mitochondrial transition pore and the loss of mitochondrial function [207]. Even more so, at a concentration of ~ 1.8 mM Ca2+ (characteristic of blood), there is no mechanism to prevent the mitochondrial transition pore from opening [209]. The study of Bertero et al. showed that when 1 mM Ca2+ was added to a calcium-free respiration buffer, mitochondria derived from skeletal muscle slightly and transiently accelerated oxygen consumption and depolarized within 20 s [210]. However, various independent studies have demonstrated that exogenous mitochondria are absorbed and functionally integrated into recipient cells in a 1.8 mM Ca2+ environment [124, 125, 211]. The following are likely reasons: (1) Isolated mitochondria are rapidly internalized by recipient cells without prolonged exposure to calcium-rich extracellular environments [124, 192]; (2) Mitochondrial Ca2+ uniporter (MCU) loses function during transfer. A study showed that when mitochondria were preincubated with ruthenium red, an MCU inhibitor, the high calcium status resulted in partial depolarization of mitochondria and did not affect mitochondrial respiration [210]; and (3) Mitochondria are damaged in the extracellular environment but reinternalized and utilized in recipient cells. As found in spontaneous mitochondrial transfer, damaged mitochondria also migrate to other cells and promote mitochondrial biogenesis of the recipient cell to enhance the function of the recipient cell [93].

To solve the integrity problems after mitochondrial isolation and the challenges of exposure to the extracellular environment, perhaps future research should focus more on spontaneous mitochondrial transfer. When cells transfer mitochondria through TNTs, EVs, GJCs, or cell fusion, they wrap mitochondria in a cytoplasmic environment through cell membranes to avoid interference by complicated extracellular environments. Flow cytometry and proteomics data suggest that some cell-free mitochondria found in blood are enclosed by EVs [212]. Therefore, mitochondria can be wrapped with liposomes and vesicles for transfer in future studies. In fact, such manipulations have been reported in recent studies [132, 213]. Moreover, Picone et al. used synaptosomes to vehicle and transfer functional mitochondria, which preserved mitochondria for a longer time and even allowed them to undergo cryopreservation [49].

In addition, using stem cells with strong mitochondrial transferability for transplantation is also a feasible option. Huang et al. used iron oxide nanoparticles to pretreat hMSCs to overexpress CX43 in hMSCs, thereby significantly increasing the mitochondrial transfer efficiency of hMSCs to damaged injured alveolar epithelial cells [214]. Tseng et al. overexpressed Miro1 in MSCs to enhance the metabolic benefits of mitochondrial transfer after neuronal oxidative damage, and the benefits may be due to the enhancement of the transfer efficiency [215]. As the advantages of editing cells to mediate efficient mitochondrial transfer become prominent and the effects of treatment are gradually elucidated, using engineered cells to provide mitochondrial replenishment may be a promising strategy in the future.

### Exogenous mitochondrial compatibility and retention

One of the thorny issues facing mitochondrial transfer is compatibility with nDNA. More compatible mtDNA-nDNA pairing and better mtDNA-nDNA metabolic profile pairing had better effects on cell proliferation [68, 216]. Low levels of mtDNA-nDNA compatibility severely affect the reprogramming efficiency of cells [68, 217]. The existing AMT/T tool "Mitopunch" can form a variety of mtDNA and nDNA pairs, expanding the genomic combinations in the eukaryotic kingdom and increasing the opportunities for addressing the secret of mtDNA-nDNA compatibility [68]. However, mitochondria interact not only with nDNA but also physically and functionally with other organelles in the cell [218]. These contacts are critical to organelle function and overall cellular homeostasis, and dysfunction of these contacts can lead to disease [121]. Therefore, it is also crucial to establish an excellent functional connection between exogenous mitochondria and the organelles of the recipient cell.

The retention time of mitochondria also needs attention. It should be noted that the transfer methods in different studies are not uniform, resulting in the retention time of mitochondria in recipient cells being very different. Prolonging the mitochondrial retention time can undoubtedly enhance the clinical therapeutic effect and avoid repeated administration. The entry point to study this issue currently is the compatibility between mitochondria and DNA. The mismatch of nDNA is most likely to reduce mitochondrial gene transcription and thus reduce the total number of mitochondria in cells. However, nDNA mimatch cannot explain the complete clearance of exogenous mitochondria. Mitochondrial degradation mainly depends on mitophagy, while mitochondrial fusion enables efficient exchange and mixing of contents between different mitochondria, blocking mitochondrial degradation [219, 220]. After entering the recipient cell, whether the exogenous mitochondria can fuse well with the endogenous mitochondria, whether the autophagy of the recipient cell or the mitochondria autophagy is changed, and whether the cell selectively autophagy of the exogenous mitochondria are some of the keys for subsequent research.

### Safety of mitochondrial transfer/transplantation: is more always better?

Due to conditions limitations, many kinds of research on mitochondrial transfer are based on the levels determined in in vitro studies, which cannot completely simulate the environment of cells in vivo and the communication between various cells. Although many studies suggest that mitochondria can be targeted for transfer to damaged cells in the body, other cells still have the possibility of absorption, which may cause unnecessary cell activation. Phinney et al. showed that when MSCs transfer mitochondria to macrophages, microRNA-containing exosomes are also released to inhibit macrophage activation via negative regulators of Toll-like receptor signalling [109]. At this stage, AMT/T obviously cannot meet this demand. Mitochondrial transfer/transplantation has also been found to promote tumorigenesis, drug resistance, and sensitivity to chemotherapy in the treatment of tumours. Therefore, more screening is needed for the treatment subjects of mitochondrial transfer, and it needs to be considered whether mitochondrial transfer promotes abnormal cell activation and proliferation.

Moreover, mitochondria are involved in various cell death modalities and are viewed as sensors and amplifiers of cell death [221]. More dense mitochondria may mean detrimental ROS amplification and more cytochrome C produced when cells are exposed to a harsh environment, leading to insufficient cell clearance and cell apoptosis [222]. On the other hand, research has shown that obtained mitochondria can promote an increase in endogenous mtDNA concentration [62]. Superfluous mitochondria may lead to extra ATP production, which exceeds the systemic/organic energy demand and adversely affects the organism [223, 224]. A classic drug for the treatment of diabetes, metformin, exerts its pharmacological effects by inhibiting mitochondrial ETC, and has been implicated in treating various diseases and tumour suppression [225–227]. Indeed, mitochondria maintain their homeostasis through continuous biogenesis and mitophagy, thus maintaining energy homeostasis. However, whether mitochondrial transfer/transplantation affects mitochondrial homeostasis in recipient cells has not been clearly described in the literature. More attention should be given to the quantity and quality of mitochondria and their effects on mitochondrial homeostasis during transfer/transplantation.

## Concluding remarks

Mitochondrial transfer and mitochondrial transplantation are concepts that have attained ample attention in recent years. It makes sense to consider these two subjects together since they cover common topics such as mitochondrial donor selection, the mechanism by which mitochondria enter recipient cells, and the effects that mitochondria exert. As described in this review, the exploration of spontaneous mitochondrial transfer provides a theoretical basis for the future treatment of diseases by mitochondrial transplantation. Although mitochondrial transfer/transplantation mechanisms are currently not well understood, these processes still show great therapeutic potential. Thus, extensive and normative experiments should be performed to understand the molecular and cellular mechanisms of mitochondrial transfer/transplantation and the demonstrate their efficacy, which will serve as the foundation for future clinical trials.

## Data Availability

Not applicable.

## Abbreviations

AMT/T: Artificial mitochondrial transfer/transplantation
AD: Alzheimer’s disease
BMSCs: Bone marrow mesenchymal stem cells
cADPR: Cyclic ADP-ribose
CAFs: Cancer-associated fibroblasts
CECs: Corneal epithelial cells
CPEO: Chronic progressive external ophthalmoplegia
CXCL1/8: C-X-C ligand 1/8
CXs: Connexins
DAMPs: Damage-associated molecular patterns
DI: Direct injection
ECs: Endothelial cells
EMCs: Endometrial gland‐derived mesenchymal cells
ETC: Electron transfer chain
EVs: Extracellular vesicles
GBM: Glioblastoma
GSLC: GBM stem-like cells
GJCs: Gap junction channels
HCs: Hemichannels
hMADS: Human multipotent adipose-derived stem cells
HO-1: Heme oxygenase-1
i.c.v. injection: Intracerebroventricular injection
i.v. injection: Intravenous injection
iPSC-MSC: Induced pluripotent stem cell-derived MSCs
IP-TNTs: Interpericyte tunneling nanotubes
IR: Ischaemia–reperfusion
LHON: Leber’s hereditary optic neuropathy
MCU: Mitochondrial Ca2+ uniporter
MELAS: Mitochondrial encephalopathy lactic acidosis and stroke-like episodes
MERRF: Myoclonus epilepsy associated with ragged-red fibers
Mfn2: Mitofusin 2
MIDD: Maternally inherited diabetes and deafness
MMSCs: Mesenchymal multipotent stromal cells
MOM: Mitochondrial outer membrane
MRC-5: Human embryonic lung fibroblasts
MSCs: Mesencgymal stem cells
mtDNA: Mitochondrial DNA
nDNA: Nuclear DNA
NF-κB: Transcription factor nuclear factor kappa B
NMCs: Neonatal mice cardiomyocytes
NSCs: Neural stem cells
OGD: Oxygen-glucose deprivation
OPA1: Optic atrophy 1
OXPHOS: Oxidative phosphorylation
PCA: Prostate cancer
PD: Parkinson’s disease
ROS: Reactive oxygen species
RyRs: Ryanodine receptors
Sig-1R: Sigma-1 receptor
SIMR: Stable isolated mitochondrial recipient
T-ALL: T cell acute lymphoblastic leukemia
TME: Tumor microenvironment
TMs: Tumor microtubes
TNF-α: Tumor necrosis factor-α
TNTs: Tunneling nanotubes
UVR: Ultraviolet radiation
WJMSCs: Wharton’s jelly mesenchymal stem cells
